# Bioinformatics and systems biology approach to identify the pathogenetic link of neurological pain and major depressive disorder

**DOI:** 10.3389/ebm.2024.10129

**Published:** 2024-06-27

**Authors:** Jinjing Hu, Jia Fu, Yuxin Cai, Shuping Chen, Mengjian Qu, Lisha Zhang, Weichao Fan, Ziyi Wang, Qing Zeng, Jihua Zou

**Affiliations:** ^1^ Department of Rehabilitation Medicine, Zhujiang Hospital, Southern Medical University, Guangzhou, China; ^2^ School of Rehabilitation Medicine, Southern Medical University, Guangzhou, China; ^3^ Department of Rehabilitation Sciences, Hong Kong Polytechnic University, Kowloon, Hong Kong SAR, China; ^4^ Department of Rehabilitation, The First Affiliated Hospital, Hengyang Medical School, University of South China, Hengyang, China; ^5^ Rehabilitation Laboratory, The First Affiliated Hospital, Hengyang Medical School, University of South China, Hengyang, China; ^6^ Faculty of Health and Social Sciences, Hong Kong Polytechnic University, Kowloon, Hong Kong SAR, China; ^7^ Department of Clinical Medicine, Suzhou Vocational Health College, Suzhou, China; ^8^ The First School of Clinical Medicine, Southern Medical University, Guangzhou, China

**Keywords:** neurological pain, gene expression omnibus (GEO), major depressive disorder (MDD), bioinformatics, receiver operating characteristic (ROC)

## Abstract

Neurological pain (NP) is always accompanied by symptoms of depression, which seriously affects physical and mental health. In this study, we identified the common hub genes (Co-hub genes) and related immune cells of NP and major depressive disorder (MDD) to determine whether they have common pathological and molecular mechanisms. NP and MDD expression data was downloaded from the Gene Expression Omnibus (GEO) database. Common differentially expressed genes (Co-DEGs) for NP and MDD were extracted and the hub genes and hub nodes were mined. Co-DEGs, hub genes, and hub nodes were analyzed for Gene Ontology (GO) and Kyoto Encyclopedia of Genes and Genomes (KEGG) enrichment. Finally, the hub nodes, and genes were analyzed to obtain Co-hub genes. We plotted Receiver operating characteristic (ROC) curves to evaluate the diagnostic impact of the Co-hub genes on MDD and NP. We also identified the immune-infiltrating cell component by ssGSEA and analyzed the relationship. For the GO and KEGG enrichment analyses, 93 Co-DEGs were associated with biological processes (BP), such as fibrinolysis, cell composition (CC), such as tertiary granules, and pathways, such as complement, and coagulation cascades. A differential gene expression analysis revealed significant differences between the Co-hub genes ANGPT2, MMP9, PLAU, and TIMP2. There was some accuracy in the diagnosis of NP based on the expression of ANGPT2 and MMP9. Analysis of differences in the immune cell components indicated an abundance of activated dendritic cells, effector memory CD8^+^ T cells, memory B cells, and regulatory T cells in both groups, which were statistically significant. In summary, we identified 6 Co-hub genes and 4 immune cell types related to NP and MDD. Further studies are needed to determine the role of these genes and immune cells as potential diagnostic markers or therapeutic targets in NP and MDD.

## Impact statement

There is a close relationship between Neuropathic Pain (NP) and major depressive disorder (MDD). The underlying molecular pathology of NP and MDD is complex and drug treatments have not yielded satisfactory results, thus further studies are needed to identify biomarkers and therapeutic targets. In this study, we identified common differentially expressed genes (Co-DEGs) for NP and MDD using several available datasets. Co-DEGs, hub genes, and hub nodes were analyzed for GO and KEGG enrichment. We also identified the immune-infiltrating cell component by ssGSEA and analyzed the relationship. We identified 6 co-hub genes, which included ANGPT2, EPO, HGF, MMP9, PLAU, and TIMP2. There were also significant differences in the abundance of activated dendritic cells, effector memory CD8 T cells, memory B cells, and regulatory T cells. Overall, this study may lead to new diagnostic markers and/or therapeutic targets for NP and MDD diseases.

## Introduction

Neuropathic Pain (NP) is caused by a somatic sensory neurological condition and may be divided into central and peripheral NP [1, 2]. The prevalence of NP accounts for 6.9%–10.0% of the general population and significantly affects physical and mental health [3]. However, the underlying mechanism of NP is complex and clinical drug treatment has not yet achieved satisfactory results, thus further in-depth exploration of NP is needed [4, 5].

There is a close relationship between NP and major depressive disorder (MDD) [6]. Most patients with NP also have depression and NP may promote adaptive changes in the expression of depression-related brain network genes [7]. Identifying the common pathological factors for NP and depression comorbidities will help to identify effective treatments. Some studies that have examined NP and MDD have shown that synaptic plasticity and the synaptic microenvironment may be important to the pathogenesis of NP and depression [8], where plastic changes in corticolimbic structures have been shown to be a consequence of the association of emotion with painful stimuli [9]. Additionally, the functional and structural changes in neurons due to this neural plasticity may in turn serve as biomarkers of NP [8]. Growing evidence indicates that neuroinflammation is closely related to both NP and depression [10, 11], and that immune system abnormalities mediated by cytokines are strongly linked to the development of NP [10, 12]. Increasing neurodifferentiation and restoring the typical morphology of neonatal dendrites may improve depression-like symptoms in NP [13]. In addition, NP-induced emotional disorders are associated with hippocampal (HC) neuroinflammation [14], whereas abnormal regulation of HC dendritic spines may explain the link between chronic NP and depression [15, 16]. A potential therapeutic focus for managing complicated depressive symptoms in NPs may be the LA/BLA-CeA synapse in the amygdala [17]. Glial cells significantly affect synaptic plasticity and have a significant impact on the progression of coexisting NP and depression [18]. Regulating the P2X7-ROS signaling pathway to inhibit ferroptosis in spinal cord microglia alleviates rats with pain and depressive behavioral changes [19]. Although some progress has been made, much remains unclear regarding the co-pathogenesis of NP and depression co-morbidities. Therefore, an in-depth study of the relationship between these two diseases is needed to identify effective treatments.

We hypothesize that in the pathogenesis of NP and MDD, the expression and activity of specific co-hub genes and immune cells may exhibit significant differences between disease and healthy states, and may play a key role in the common pathological processes of these two diseases. Identifying diagnostic markers and treatment targets for NP and MDD will increase our understanding of the relationship between these two diseases and guide future clinical practice and scientific research. It will also assist doctors in accurately diagnosing NP and MDD comorbidities and provide more effective treatment options for patients. A biomarker is a measurable indicator of a biological state, providing information about disease prognosis and progression [20]. Bioinformatics will facilitate the identification of the co-occurrence mechanism of NP and depression and identify potential biomarkers and prognostic indicators to accurately diagnose and treat NP and depression comorbidities. A recent analysis screened a set of genes associated with NP-induced depression; however, it was done with a single dataset associated with a high false-positive rate [21]. Small sample sizes and different microarray platforms can introduce significant bias in the results. Therefore, finding new therapeutic targets and robust diagnostic markers is required. In this study, we screened for Co-hub genes between NP and MDD by integrating 4 databases and identified the immune cells associated with the co-morbidity of NP and MDD by analyzing the differences in the immune cell signatures.

## Materials and methods

### Data downloads

The Gene Expression Omnibus (GEO) is a public repository that archives and distributes high-throughput gene expression and other functional genomics data sets, with web-based tools for data visualization and analysis [22]. We downloaded the gene expression datasets, GSE98793 [23] and GSE32280 [24], which are associated with major depressive disorder (MDD) patients from the GEO database using the R package GEOquery [25] The species source for both the GSE98793 and GSE32280 datasets was *Homo sapiens* and the data platform was GPL570 [HG-U133_Plus_2] Affymetrix Human Genome U133 Plus 2.0 Array. We also downloaded the GSE24982 [26] and GSE30691 [27] gene expression profiles of Neuropathic Pain (NP) patients, in which the species source for both was *Rattus norvegicus*. The data platform for the GSE24982 dataset was GPL1355 [Rat230_2] Affymetrix Rat Genome 230 2.0 Array, whereas the data platform for the GSE30691 dataset was the GPL85 [RG_U34A] Affymetrix Rat Genome U34 Array.

The chip GPL platform file was used for all dataset probe name annotations. We selected data from 128 patients with major depressive disorder (MDD) (MDD group, group: MDD) and 64 healthy controls (control group, group: Control) from the GSE98793 datasets of whole blood samples for inclusion in the analysis. A total of 16 subjects from the GSE32280 datasets were analyzed, including 8 examples of peripheral blood lymphocytes from matched healthy controls (control group, group: Control) and 8 peripheral blood lymphocyte samples from MDD patients. In addition, in the Neuropathic Pain (NP) dataset, GSE24982, we used a total of 40 mRNA data samples based on the L4 and L5 Dorsal Root Ganglion (DRG), including 20 control (group: Control) mRNA samples and 20 mRNA samples from the spinal nerve ligation model of neuropathic pain in rats (NP group, group: NP). The GSE30691 dataset contains mRNA samples from 11 rat neuropathic pain spinal nerve ligation (Ch) models (NP group, group: NP) and 9 Sham (SH) control (group: Control) mRNA data samples. The specific grouping of the information from these datasets is listed in Table 1. The datasets we selected are all expression profiling by array, and the sample size is sufficient to meet our analysis requirements. In addition, to ensure that our control samples match the experimental conditions, the healthy control samples and diseased samples in these datasets are as consistent as possible in terms of sample collection methods, time, and other experimental conditions to minimize the impact of confounding factors.

**TABLE 1:** List of GEO datasets Information.

	GSE98793	GSE32280	GSE24982	GSE30691
Platform	GPL570	GPL570	GPL1355	GPL85
Species	*Homo sapiens*	*Homo sapiens*	*Rattus norvegicus*	*Rattus norvegicus*
Tissue	whole blood	peripheral blood lymphocytes	L4 and L5 Dorsal Root Ganglion	Dorsal Root Ganglion
Samples in Case group	128	8	20	11
Samples in Control group	64	8	20	9
Reference	Replicable and Coupled Changes in Innate and Adaptive Immune Gene Expression in Two Case-Control Studies of Blood Microarrays in Major Depressive Disorder	Blood-based gene expression profiles models for classification of subsyndromal symptomatic depression and major depressive disorder	Dynamic changes in the microRNA expression profile reveal multiple regulatory mechanisms in the spinal nerve ligation model of neuropathic pain	Multiple chronic pain states are associated with a common amino acid-changing allele in KCNS1

GEO, gene expression omnibus; MDD, major depressive disorder; NP, neuropathic pain.

### Editing of raw data and differential gene analysis

The MDD (GSE98793, GSE32280) and NP (GSE24982, GSE30691) datasets were combined for analysis. To minimize the variance in sample combinations across batches, we first standardized internally using the normalizeBetweenArrays function of the R limma package with the quantile normalization method [28]. We corrected the combined data for batch effects using the remove batch effect function, which enabled us to obtain the combined MDD and NP datasets. The MDD datasets contained 136 cases (disease group, group: Case/MDD) and 72 controls (control group, group: Control). The NP datasets contained 31 cases (disease group, group: Case/NP) and 29 controls (control group, group: Control). The expression values for the samples in the MDD and NP datasets were then analyzed using Principal Component Analysis (PCA) before and after correction [29].

Differentially expressed genes (DEGs) are a subset of genes that express differently among experimental conditions, used to determine biological functions or predict therapeutic outcomes. To identify the potential mechanism of action of DEGs in MDD and NP and the associated biological features and pathways, we performed a differential expression analysis on the case and control groups using the R limma package. Genes that met the criteria |logFC| >0 and *p*-value <0.05 were considered DEGs for subsequent studies. |logFC| represents the absolute log2 value of the fold change in gene expression. To obtain the common differentially expressed genes (Co-DEGs) associated with MDD and NP, we selected the DEGs from the differential analysis of the MDD and NP datasets DEGs and constructed a Venn diagram. A volcano plot was generated using ggplot2 of the R package and a heat map was used to display the results.

### Protein-protein interaction network analysis

A protein-protein interaction (PPI) network is made up of different proteins that engage with one another to function in a variety of biological processes, including signaling, regulation of gene expression, substance metabolism, energy production, and cell cycle regulation. A database for identifying known proteins and predicting protein interactions is the STRING database [30]. We constructed a PPI network of Co-DEGs linked to both NP and MDD disease [minimum required interaction score: middle confidence (0.400)], which was visualized with Cytoscape [31] (version 3.9.1). Using the MCODE plugin, we mined the hub nodes with connections to other PPI network nodes (K score: 2, Cutoff degree: 2, Cutoff node score: 0.2) [32]. These nodes were highly interconnected with one another and may play a role in regulating various biological processes associated with NP and MDD.

A molecular complex with a particular biological activity may be represented by closely connected local areas in the PPI network. We also used four algorithms to mine the scores of Co-DEGs in PPI networks that are connected to other PPI network nodes, which included MCC (Maximal Clique Centrality) [33], MNC (the maximal neighborhood coefficient), EPC (edge percolated component), and Closeness. According to our rankings of the Co-DEGs, the top 20 Co-DEGs across the four algorithms were considered as hub genes (hub genes, mRNA).

### GO and KEGG enrichment analysis

Large-scale functional enrichment studies, including biological process (BP), molecular function (MF), and cellular component (CC), are frequently carried out using the Gene Ontology (GO) database [34]. The Kyoto Encyclopedia of Genes and Genomes (KEGG) is a widely used database that genomic data for biological pathways, diseases, and drugs [35]. We conducted GO and KEGG enrichment analyses for MDD and NP disease-associated Co-DEGs, PPI network hub nodes, and hub genes in the PPI network using the clusterProfiler [36]. *p* values < 0.05 and FDR values (q value) < 0.05, which were considered statistically significant as a criteria for selection and Benjamini-Hochberg was used to correct *p* values.

### Identification and correlation analysis of immune-infiltrating cells

To more precisely measure the percentage of various immune cells in samples associated with MDD and NP. Single-sample gene-set enrichment analysis (ssGSEA) was used to identify highly sensitive and specific differentiation of the various human immune cells in the tumor microenvironment (TME). The algorithm generated a set of 28 genes to mark different tumor-infiltrating resistant cell types from a study of published tumor immune infiltration articles [37, 38]. The degree of immune cell infiltration in each sample was represented by an enrichment score computed by ssGSEA in the GSVA tool of R. The varying abundance of infiltrating immune cells between the Case (MDD/NP) group and the Control group are shown using a heat map and a boxplot for the MDD and NP datasets. We then calculated the correlation between immune cells and Co-hub genes in different disease samples by combining the gene expression matrix of both disease samples. The correlation heat map was displayed using the R package heatmap.

### Co-hub gene correlation analysis and differential expression analysis

Finally, the expression of Co-hub genes in the MDD and NP datasets was examined using the Spearman method. The results of the correlation analysis were presented by plotting the correlation heat map. Next, we selected the results for the Co-hub genes with the same trend and displayed them by plotting the correlation scatter graph with the R package ggplot2.

We then established a group comparison graph for the Co-hub genes in various groups (Case/Control) of the MDD and NP datasets. The Receiver operating characteristic (ROC) Curve [39] is a composite indicator that represents continuous variables of sensitivity and specificity. We used the R package pROC program to plot the ROC curves of the Co-hub genes in the MDD and NP datasets and calculated the Area Under the Curve (AUC) of the ROC curve to determine the diagnostic significance of the Co-hub genes. The Receiver operating characteristic (ROC) curve AUC typically falls between 0.5 and 1. The diagnostic impact increases as the AUC gets near 1. The AUC exhibits low accuracy in the range of 0.5–0.7, some accuracy in the range of 0.7–0.9, and high accuracy in the range of 0.9 or above.

### Statistical analysis

R software was used for data processing and analysis in this paper (Version 4.2.2). We calculated the normally distributed variables using an independent Student t-test to compare two sets of continuous variables. We used the Mann-Whitney *U* test to examine differences among factors with non-normal distributions (i.e., Wilcoxon rank sum test). If not explicitly indicated, a Spearman correlation was used to calculate the correlation coefficients between different molecules. The *p* values were two-sided with *p* < 0.05 being the threshold for statistical significance.

## Results

### Dataset pre-processing and differential gene analysis

**FIGURE 1:**
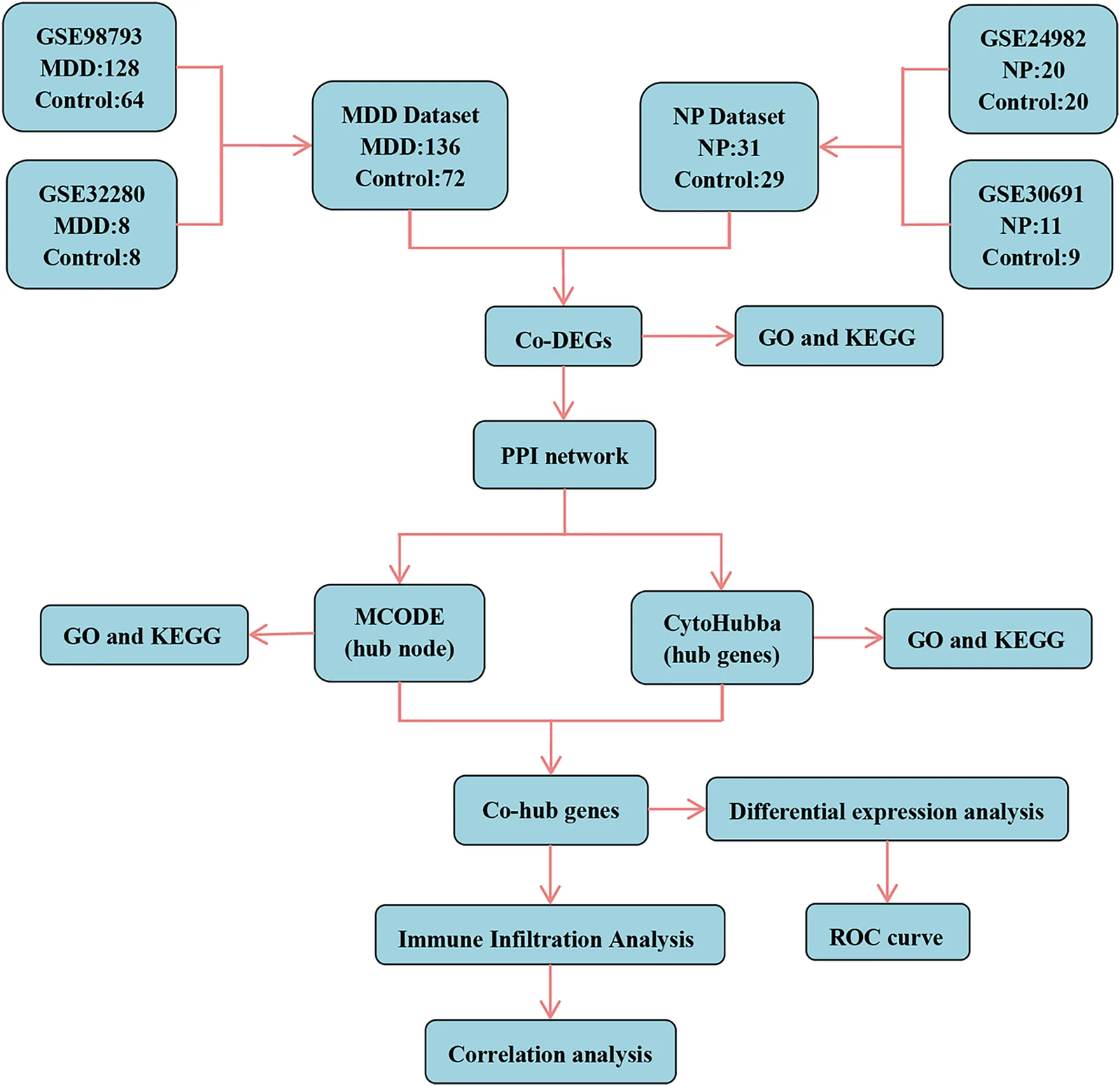
Technology roadmap. MDD, major depressive disorder; NP, Neuropathic Pain; Co-DEGs, Common differentially expressed genes; PPI network, Protein-protein interaction network; MCODE, Molecular Complex Detection; GO, Gene Ontology; KEGG, Kyoto Encyclopedia of Genes and Genomes; Co-hub genes, Common hub genes; ROC, Receiver operating characteristic curve.

**FIGURE 2:**
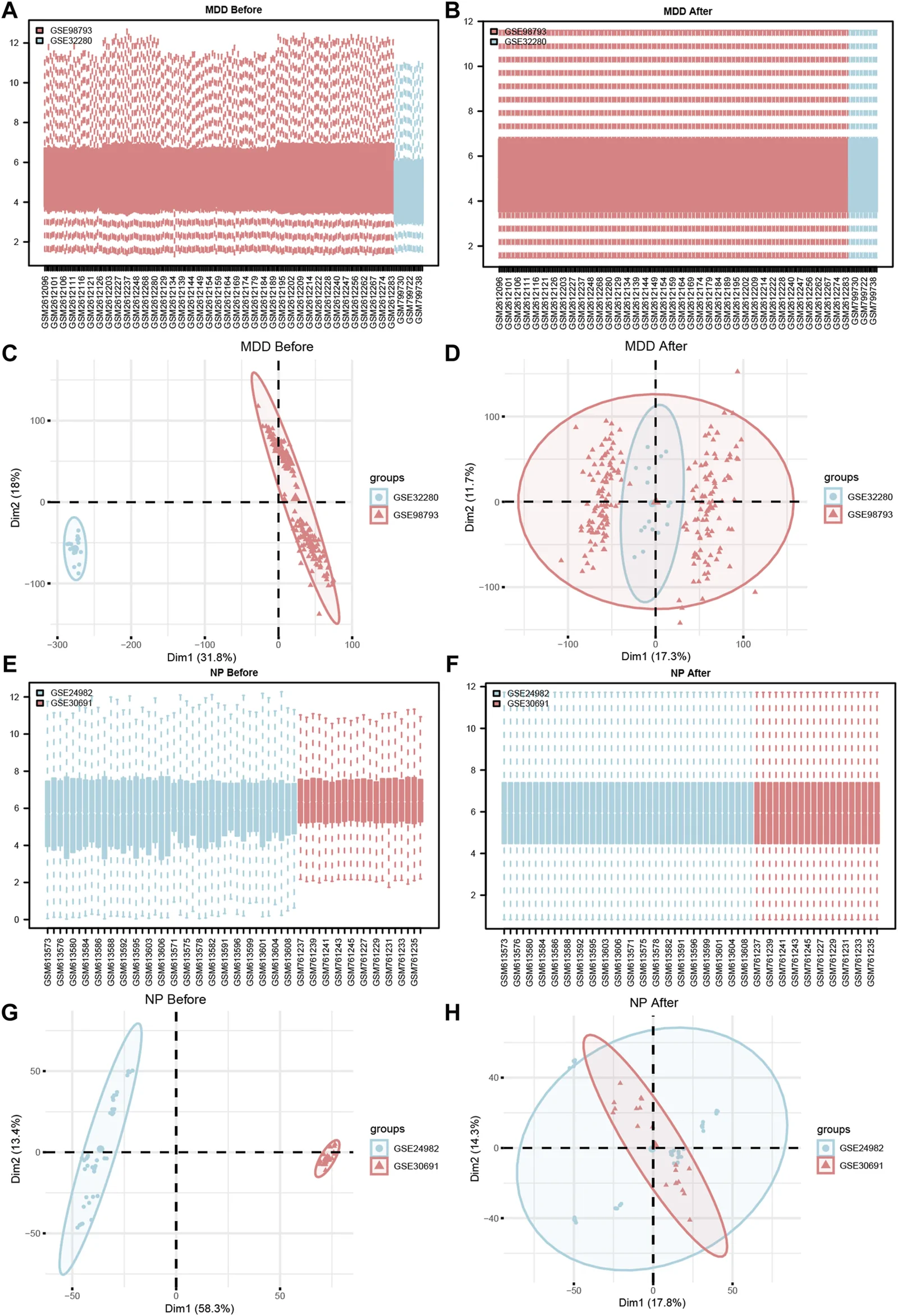
Datasets de-batch processing. **(A,B)** The boxplot before **(A)** and after **(B)** the MDD datasets removes the batch effect treatment. **(C,D)** The PCA plots before **(C)** and after **(D)** of the MDD datasets removes the batch effect treatment. **(E,F)** The boxplot before **(E)** and after **(F)** of the NP datasets removes the batch effect treatment. **(G,H)** The PCA plots before **(G)** and after **(H)** of the NP datasets removes the batch effect treatment. MDD, major depressive disorder; NP, Neuropathic Pain; PCA, Principal Component Analysis.

The technical route of this study is shown in Figure 1. Using the ControlizeBetweenArrays function of the limma package, we normalized the two major MDD datasets (GSE98793 and GSE32280) and the two NP datasets (GSE24982 and GSE30691), respectively. The MDD datasets (Figures 2A, B) and the NP datasets (Figures 2E, F) were obtained through a batch effect correction of the combined data using the “remove batch effect” function. The MDD datasets include 136 MDD samples (group: MDD) and 72 control samples (group: Control). The NP datasets consist of 31 NP samples (group: NP) and 29 control samples (group: Control). In addition, to convert mouse to human genes for subsequent analysis, the R package homologene was used to conduct an ID transformation of the NP datasets.

To verify the effect of removing the batch effect (Figures 2C, D, G, H), we grouped the MDD, and NP datasets according to the source of the samples. For the dataset expression matrix, before, and after the batch effect was eliminated, we performed a Principal Component Analysis (PCA). The results indicated that after the batch removal process, the batch effect was essentially eliminated from the MDD and NP datasets.

**FIGURE 3:**
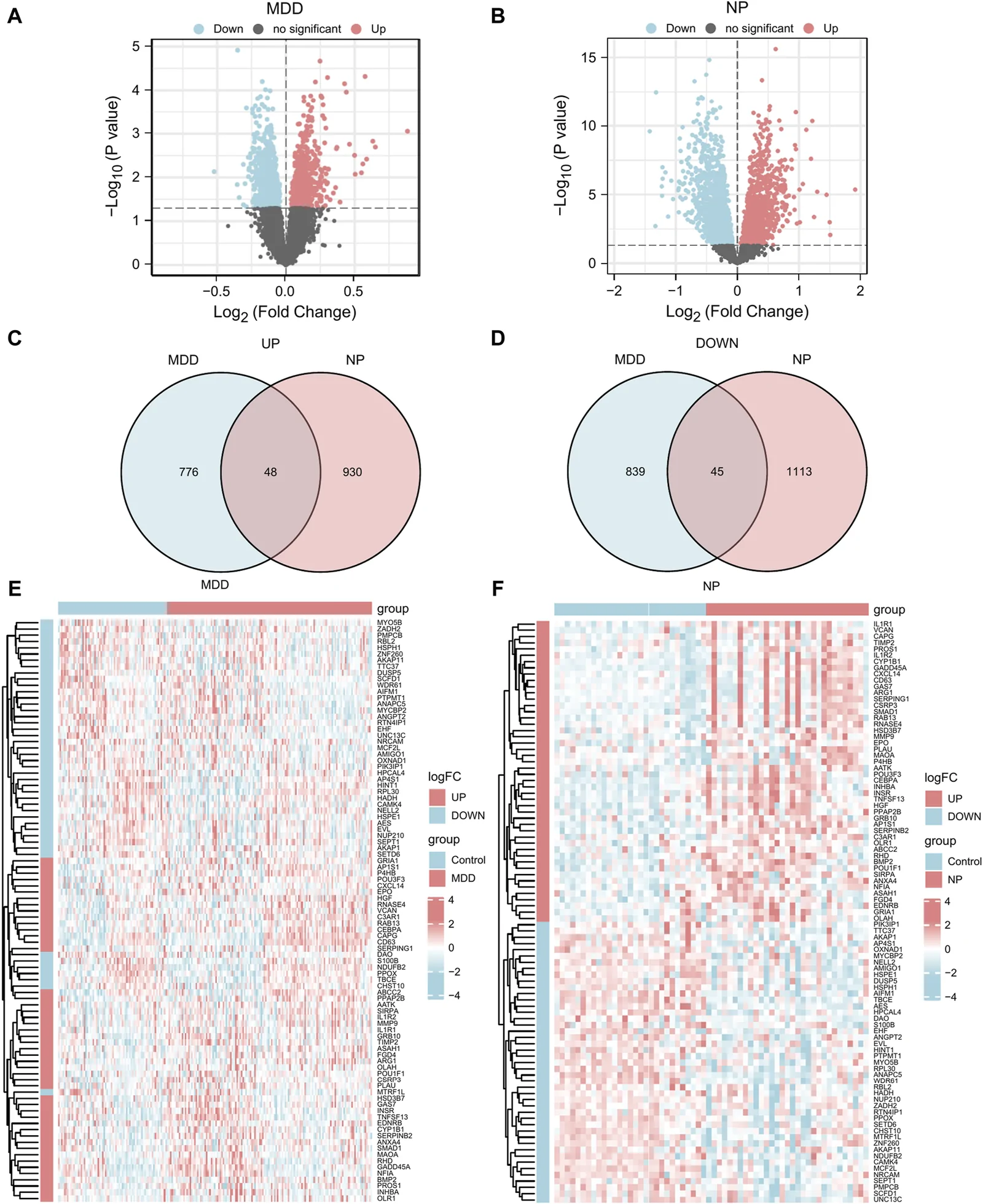
DEGs analysis of MDD datasets and NP datasets. **(A,B)** The volcano map of DEGs analysis between the disease group (group: Case/MDD/NP) and control group (group: Control) in the MDD datasets **(A)** and NP datasets **(B)**. **(C,D)** The Venn map of the up-regulation **(C)** and down-regulation **(D)** DEGs in the MDD and NP datasets. **(E,F)** The complex numerical heat map of Co-DEGs in the MDD datasets **(E)** and NP datasets **(F)**. MDD, major depressive disorder; NP, Neuropathic Pain; DEGs, differentially expressed genes; Co-DEGs, Common differentially expressed genes.

Following Principal Component Analysis (PCA), we obtained DEGs between different groups of the MDD and NP datasets. The MDD datasets yielded 21,655 DEGs, of which 1708 met the criteria. For the Case/MDD group, there were 824 upregulated DEGs and 884 downregulated DEGs. The NP datasets yielded 4076 DEGs, of which 2,136 met the criteria. For the Case/NP group, 978 exhibited high expression (low expression in the Control group with positive logFC), and 1,158 genes exhibited low expression (high expression in the Control group with negative logFC). The results for the MDD and NP datasets are depicted in volcano plots (Figures 3A, B).

To obtain Co-DEGs for MDD and NP, we focused on the intersection of 48 upregulated and 45 downregulated Co-DEGs that were presented in a Venn diagram (Figures 3C, D). The data and annotation for these Co-DEGs is listed in Table 2. We examined differential expression of 93 Co-DEGs from the MDD and NP datasets in various groups. Heat maps were used to display the results of the differential analysis using the R package heatmap (Figures 3E, F). As shown in Figure 3, 93 Co-DEGs showed significant differences in expression between the groups based on the MDD and NP datasets.

**TABLE 2:** List of Common differentially expressed genes in MDD datasets and NP datasets.

Common differentially expressed genes					
UP			DOWN		
AATK	FGD4	P4HB	AES	HINT1	PPOX
ABCC2	GADD45A	PLAU	AIFM1	HPCAL4	PTPMT1
ANXA4	GAS7	POU1F1	AKAP1	HSPE1	RBL2
AP1S1	GRB10	POU3F3	AKAP11	HSPH1	RPL30
ARG1	GRIA1	PPAP2B	AMIGO1	MCF2L	RTN4IP1
ASAH1	HGF	PROS1	ANAPC5	MTRF1L	S100B
BMP2	HSD3B7	RAB13	ANGPT2	MYCBP2	SCFD1
C3AR1	IL1R1	RHD	AP4S1	MYO5B	SEPT1
CAPG	IL1R2	RNASE4	CAMK4	NDUFB2	SETD6
CD63	INHBA	SERPINB2	CHST10	NELL2	TBCE
CEBPA	INSR	SERPING1	DAO	NRCAM	TTC37
CSRP3	MAOA	SIRPA	DUSP5	NUP210	UNC13C
CXCL14	MMP9	SMAD1	EHF	OXNAD1	WDR61
CYP1B1	NFIA	TIMP2	EVL	PIK3IP1	ZADH2
EDNRB	OLAH	TNFSF13	HADH	PMPCB	ZNF260
EPO	OLR1	VCAN			

MDD, major depressive disorder; NP, neuropathic pain.

### GO and KEGG enrichment analysis of the Co-DEGs

**FIGURE 4:**
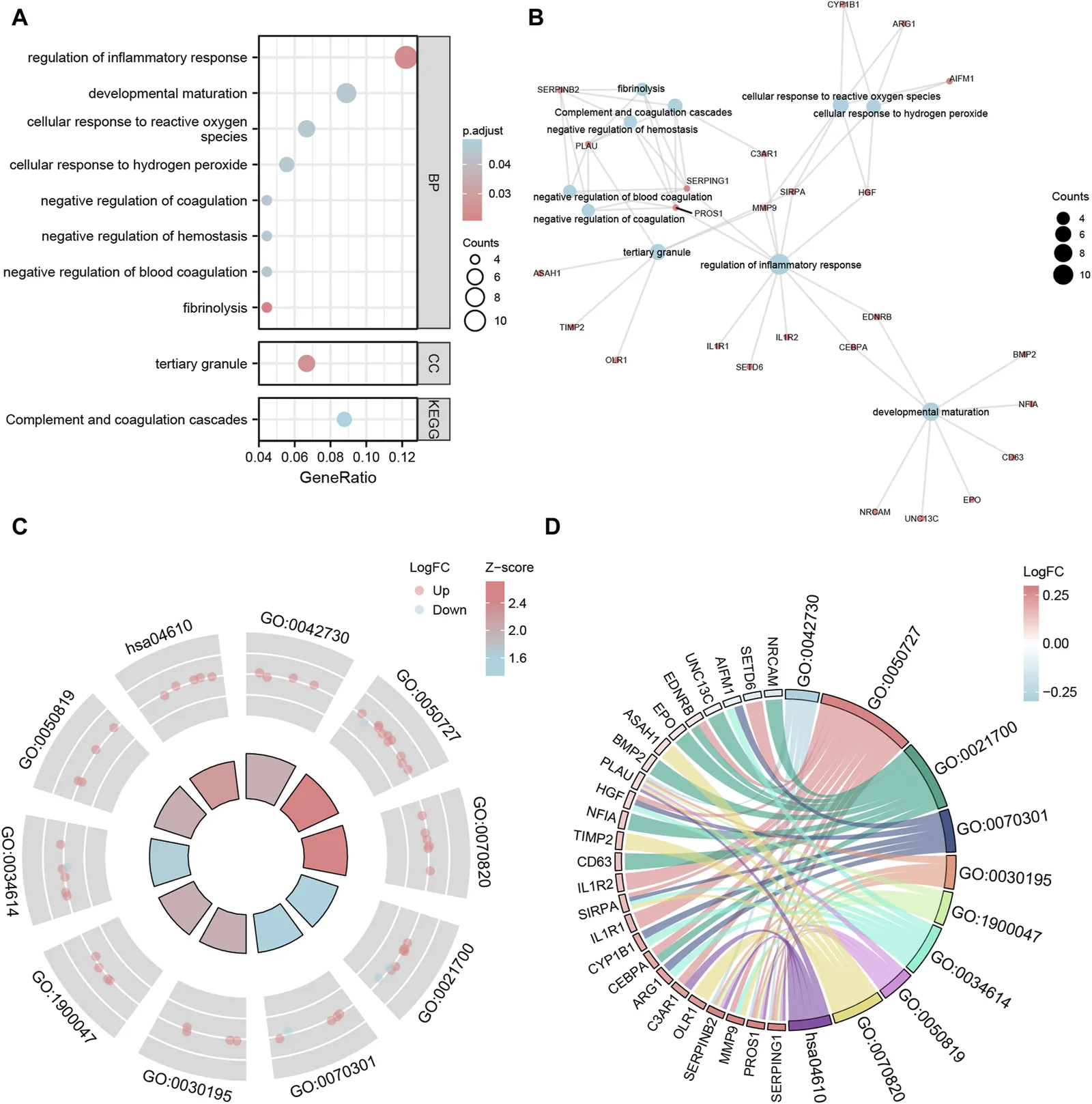
GO and KEGG enrichment analysis of Co-DEGs. **(A,B)** The bubble plot **(A)** and network plot **(B)** of GO/KEGG enrichment analysis results of Co-DEGs. **(C,D)** The circle plot **(C)** and chord plot **(D)** of GO function enrichment of Co-DEGs combined with logFC analysis results. The ordinate in the bubble chart **(A)** is GO/KEGG terms, and the length of the bubble distance to the Y axis represents the GeneRatio of GO terms. Co-DEGs, Common differentially expressed genes; GO, Gene Ontology; BP, biological process; CC: cellular component; MF: molecular function; KEGG, Kyoto Encyclopedia of Genes and Genomes. The screening criteria for GO/KEGG-enriched entries were *p* value < 0.05 and FDR value (*q*.value) < 0.05.

For the 93 Co-DEGs, we performed a functional enrichment analysis using the Gene Ontology (GO) and the Kyoto Encyclopedia of Genes and Genomes (KEGG) pathway databases (Table 3). The results indicated that 93 Co-DEGs were primarily enriched in biological processes (BP), such as fibrinolysis, regulation of inflammatory response, and developmental maturation. They were also enriched in cellular component (CC) tertiary granules and complement and coagulation cascade from the KEGG pathway database. The results are shown in the form of bubble plots and a network diagram (Figures 4A, B). We then combined the logFC values with the enrichment analysis, which generates a z score by providing the logFC values for the 93 Co-DEGs. The effects of GO and KEGG enrichment analysis by joint logFC are shown as circle plots (Figure 4C) and bubble plots (Figure 4D). The results indicated that the 93 Co-DEGs from the MDD dataset were primarily located in the BP pathway (Figures 4C, D).

**TABLE 3:** GO and KEGG enrichment analysis results of 93 Common differentially expressed genes.

Ontology	ID	Description	GeneRatio	BgRatio	P-value	p.adjust	qvalue
BP	GO:0042730	fibrinolysis	4/90	28/18,670	9.46e-06	0.021	0.018
BP	GO:0050727	regulation of inflammatory response	11/90	485/18,670	2.12e-05	0.023	0.020
BP	GO:0021700	developmental maturation	8/90	284/18,670	6.85e-05	0.045	0.040
BP	GO:0070301	cellular response to hydrogen peroxide	5/90	99/18,670	1.17e-04	0.045	0.040
BP	GO:0030195	negative regulation of blood coagulation	4/90	53/18,670	1.23e-04	0.045	0.040
BP	GO:1900047	negative regulation of hemostasis	4/90	54/18,670	1.33e-04	0.045	0.040
BP	GO:0034614	cellular response to reactive oxygen species	6/90	168/18,670	1.62e-04	0.045	0.040
BP	GO:0050819	negative regulation of coagulation	4/90	57/18,670	1.64e-04	0.045	0.040
CC	GO:0070820	tertiary granule	6/90	164/19,717	1.06e-04	0.026	0.022
KEGG	hsa04610	Complement and coagulation cascades	5/57	85/8,076	3.13e-04	0.048	0.046

GO, gene ontology; BP, biological process; CC, cellular component; MF, molecular function; KEGG, kyoto encyclopedia of genes and genomes.

### The MCODE plug-in identifies the hub nodes

**FIGURE 5:**
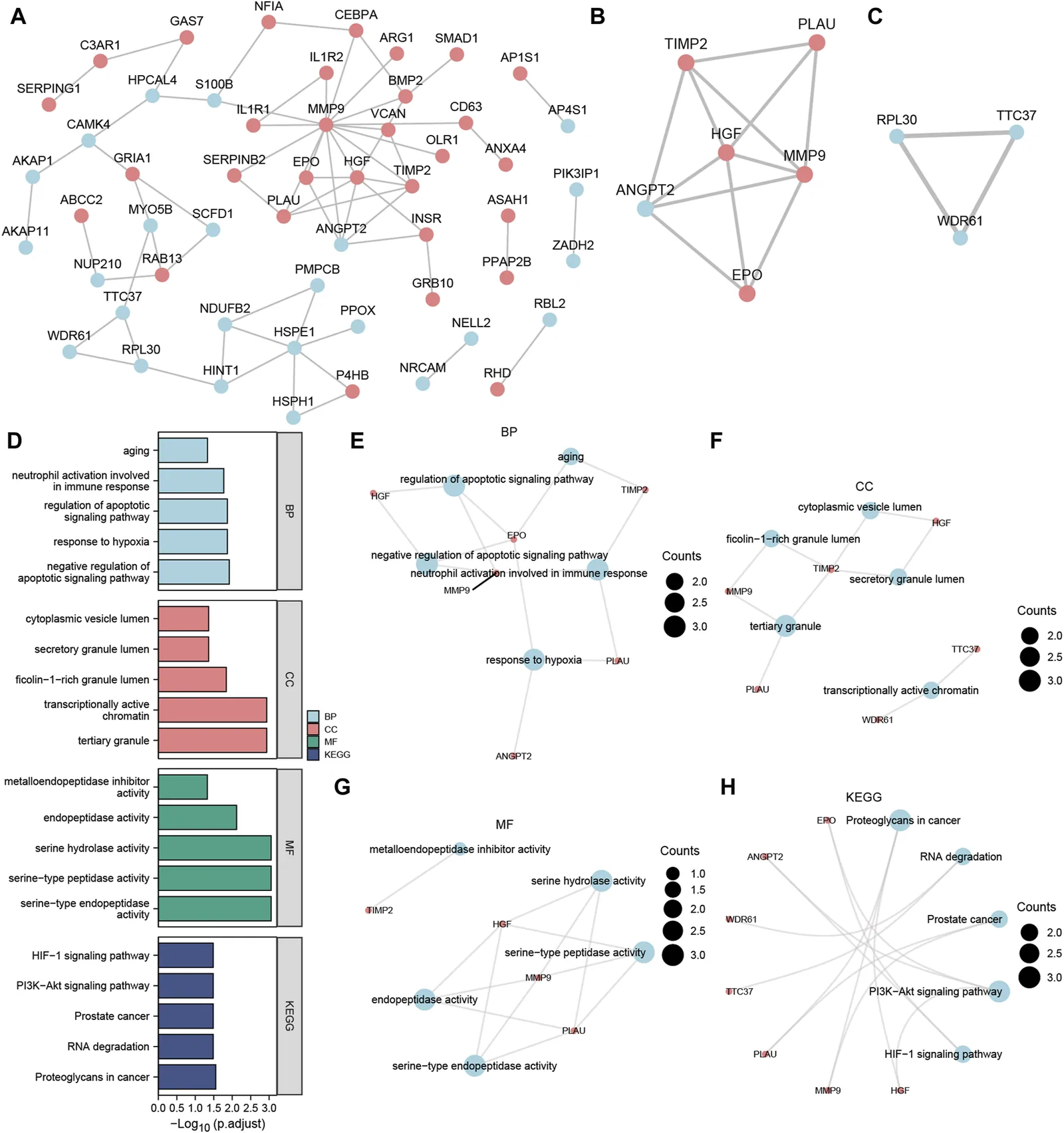
MCODE plug-in identifies the hub nodes of GO, and KEGG enrichment analysis and the PPI network. **(A)** PPI network of Co-DEGs. **(B,C)** The MCODE cluster 1 **(B)** and MCODE cluster 2 **(C)** networks of the PPI network of Co-DEGs. **(D)** Histogram of GO/KEGG enrichment analysis results of hub node. **(E–G)** The GO function enrichment analysis of Co-DEGs BP pathway **(E)**, CC pathway result **(F)**, MF pathway **(G)** result network diagram. **(H)** Ring network diagram of KEGG pathway enrichment analysis results of Co-DEGs. The ordinate in the histogram **(D)** is GO/KEGG terms, and the length of the bar distance from the Y axis represents the padj value of GO terms. Co-DEGs, Common differentially expressed genes; PPI network, Protein-protein interaction network; MCODE, Molecular Complex Detection; GO, Gene Ontology; BP, biological process; CC, cellular component; MF, molecular function; KEGG, Kyoto Encyclopedia of Genes and Genomes. The screening criteria for GO/KEGG-enriched entries were *p* value < 0.05 and FDR value (*q*.value) < 0.05.

After excluding the Co-DEGs that did not have a connection with other nodes, we constructed a PPI network (Figure 5A) consisting of 54 Co-DEGs using the STRING database. We analyzed the nodes that have connections with other nodes in the PPI network using the MCODE plugin. We then used the genes in cluster1 and cluster2 of the results as hub nodes for the Co-DEG PPI network, in which we obtained an MCODE cluster network consisting of 6 Co-DEGs (PLAU, TIMP2, HGF, ANGPT, MMP9, EPO) (Score = 4.8) (Figure 5B) and an MCODE cluster network (Score = 3) (Figure 5C) consisting of 3 Co-DEGs (RPL30, TTC37, WDR61). The 9 (hub node) genes included PLAU, TIMP2, HGF, ANGPT2, MMP9, EPO, RPL30,TTC37, and WDR61. These nodes warrant additional study as they may be important in regulating the entire BP.

On the 9 hub nodes (PLAU, TIMP2, HGF, ANGPT2,MMP9, EPO, RPL30, TTC37, WDR61), we performed GO and KEGG enrichment analyses (Supplementary Table S1). The results indicated that the BP, negative regulation of the apoptotic signaling pathway, was largely abundant in the 9 hub nodes. Enrichment in CC, such as tertiary granules, and transcriptionally active chromatin as well as MF, such as serine-type endopeptidase activity, was observed. The KEGG pathways, including proteoglycans in cancer, PI3K-Akt signaling pathway, and RNA degradation, were also enriched. The results are presented using bar graphs in Figure 5D. In addition, the GO analysis outcomes are shown for the BP pathway (Figure 5E), CC pathway (Figure 5F), and MF pathway (Figure 5G) as a network diagram, whereas the results of KEGG pathway enrichment analysis are displayed as a circular network diagram (Figure 5H).

### CytoHubba plug-in identifies the hub genes

**FIGURE 6:**
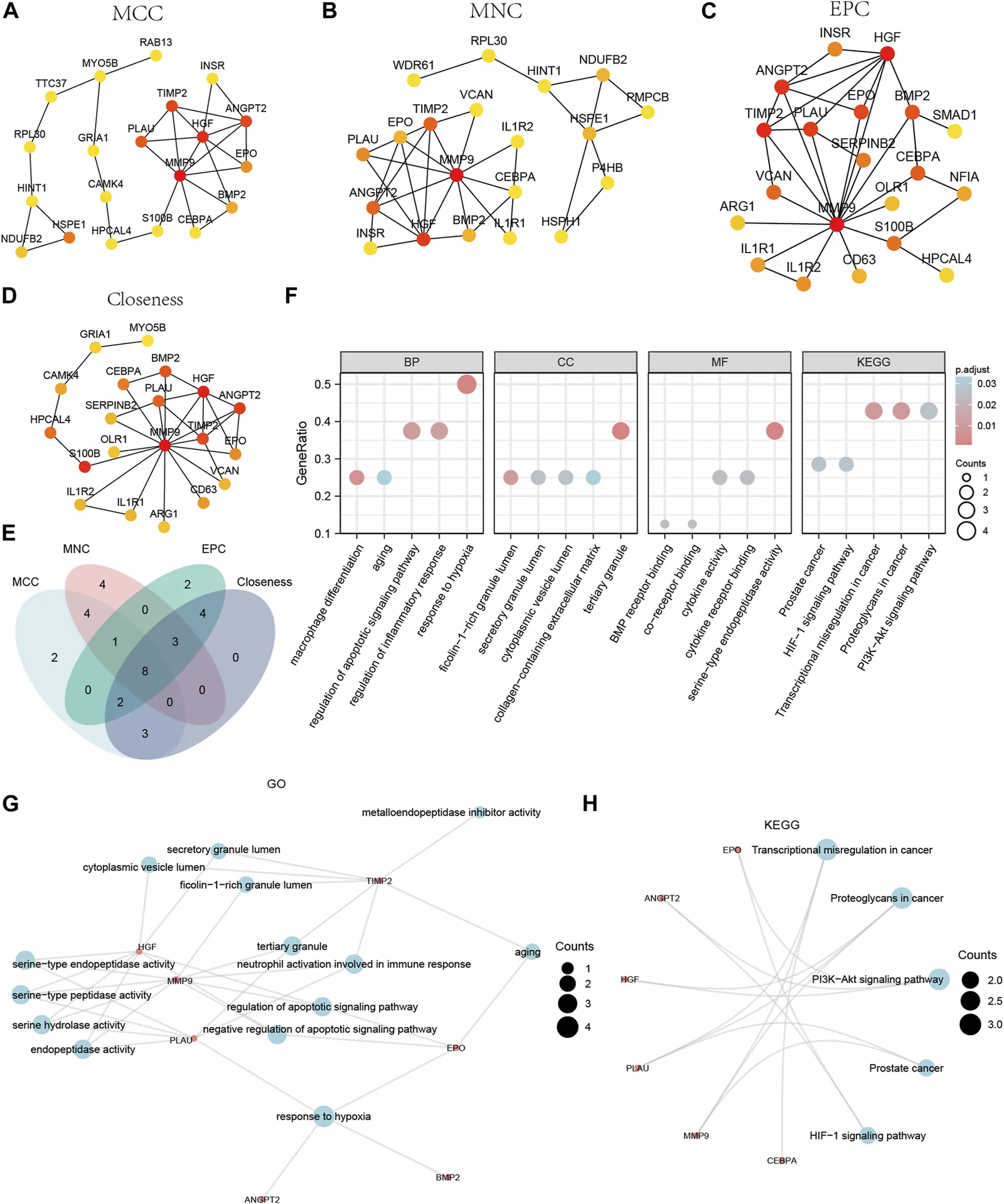
CytoHubba plug-in node network for Co-DEGs PPI network and GO and KEGG enrichment analysis. The top 20 node network of the MCC **(A)**, MNC **(B)**, EPC **(C)**, Closeness **(D)** algorithm of the PPI network of Co-DEGs. **(E)** The top 20 nodes of the Venn diagram result from the four algorithms of MCC, MNC, EPC, and Closeness in the Co-DEGs PPI network. **(F)** The bubble plot of GO/KEGG enrichment analysis results of 8 hub genes. **(G)** The network diagram of GO enrichment analysis results of Co- DEGs. **(H)** The ring network diagram of KEGG pathway enrichment analysis results of Co-DEGs. The abscissa in the bubble chart **(F)** is GO/KEGG terms, and the length of the bubble distance from the X-axis represents the GeneRatio value of GO terms. Co-DEGs, Common differentially expressed genes; PPI network, Protein-protein interaction network; MCC, Maximal Clique Centrality; MNC, the maximal neighborhood coefficient; EPC, edge percolated component; GO, Gene Ontology; BP, biological process; CC, cellular component; MF, molecular function; KEGG, Kyoto Encyclopedia of Genes and Genomes. The screening criteria for GO/KEGG-enriched entries were *p* value < 0.05 and FDR value (*q*.value) < 0.05.

 We calculated the PPI network using the cytoHubba plugin for Cytoscape using four algorithms: MCC (Maximal Clique Centrality), MNC, EPC (edge percolated component), and Closeness. The top 20 Co-DEGs with the best scores were selected to further identify the hub genes in the Co-DEGs PPI network (Figures 6A–D). The color of the dotted blocks in the graph, from yellow to red, represents a gradual increase in rating. Next, we focused on the intersection of the top 20 Co-DEGs obtained by each of the four algorithms (MCC, MNC, EPC, Closeness) to obtain the hub genes and drew Venn diagrams to display the results (Figure 6E). We obtained a total of 8 hub genes, which included ANGPT2, BMP2, CEBPA, EPO, HGF, MMP9, PLAU, and TIMP2.

We performed GO and KEGG enrichment analyses for the hub genes (Supplementary Table S2). The results indicated that the 8 hub genes were primarily enriched in BP, such as response to hypoxia. They were also enriched in CC, such as tertiary granules, and MF, such as serine-type endopeptidase activity. The KEGG pathways identified included transcriptional misregulation in cancer. We displayed the findings of the GO and KEGG enrichment analyses using bubble plots (Figure 6F). We also displayed the GO gene functional enrichment analysis findings (Figure 6G) as a network diagram and the KEGG pathway enrichment analysis results (Figure 6H) as a circular network diagram.

### The PPI network of Co-hub genes

**FIGURE 7:**
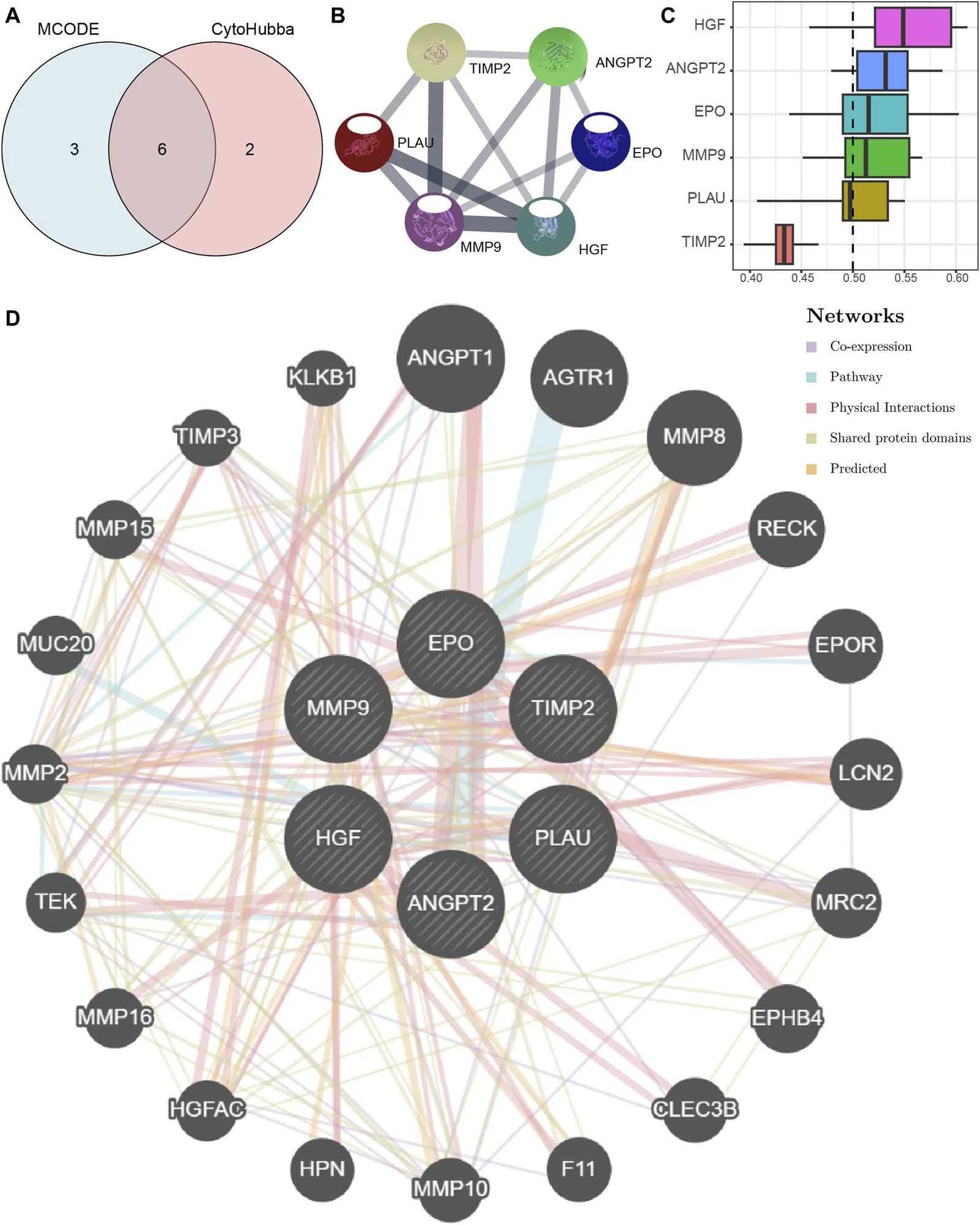
The PPI network of Co-hub genes. **(A)** The Venn diagram of the analysis results of the PPI network MCODE plugin and the cytoHubba plug-in analysis results. **(B)** The PPI network of Co-hub genes. **(C)** The histogram of functional similarity analysis results of Co-hub genes. **(D)** The PPI network of Co-hub genes based on the GeneMANIA database. PPI network, Protein-protein interaction network; MCODE, Molecular Complex Detection; Co-hub genes, Common hub genes.

We first obtained Co-hub genes by taking the intersection of the 9 hub nodes (PLAU, TIMP2, HGF, ANGPT2, MMP9, EPO, RPL30, TTC37, WDR61) and the 8 hub genes (ANGPT2, BMP2, CEBPA, EPO, HGF, MMP9, PLAU, TIMP2) (Figure 7A). As shown in Figure 7A, we obtained 6 Co-hub genes, which included ANGPT2, EPO, HGF, MMP9, PLAU, and TIMP2. We analyzed their interaction using the STRING database and visualized them with Cytoscape (Figure 7B). Next, the 6 Co-hub genes (ANGPT2, EPO, HGF, MMP9, PLAU, TIMP2) were analyzed for functional similarity. We calculated the semantic similarity among GO terms, sets of GO terms, gene products, and gene clusters using the R package GOSemSim. The results of this functional similarity analysis between the 6 Co-hub genes are presented as a boxplot in Figure 7C. HGF had the highest value of functional similarity with the other Co-hub genes among the 6 Co-hub genes.

Using the GeneMANIA database, we constructed a Co-hub gene PPI network after retaining the nodes with links to the 6 Co-hub genes (Figure 7D). As shown in Figure 7D, there are five types of interactions between the nodes of our constructed Co-hub genes PPI network and the 6 Co-hub genes, including Co-expression, Pathway, Physical Interactions, Shared protein domains, and Predicted.

### Differences in immune characteristics between the MDD and NP datasets

**FIGURE 8:**
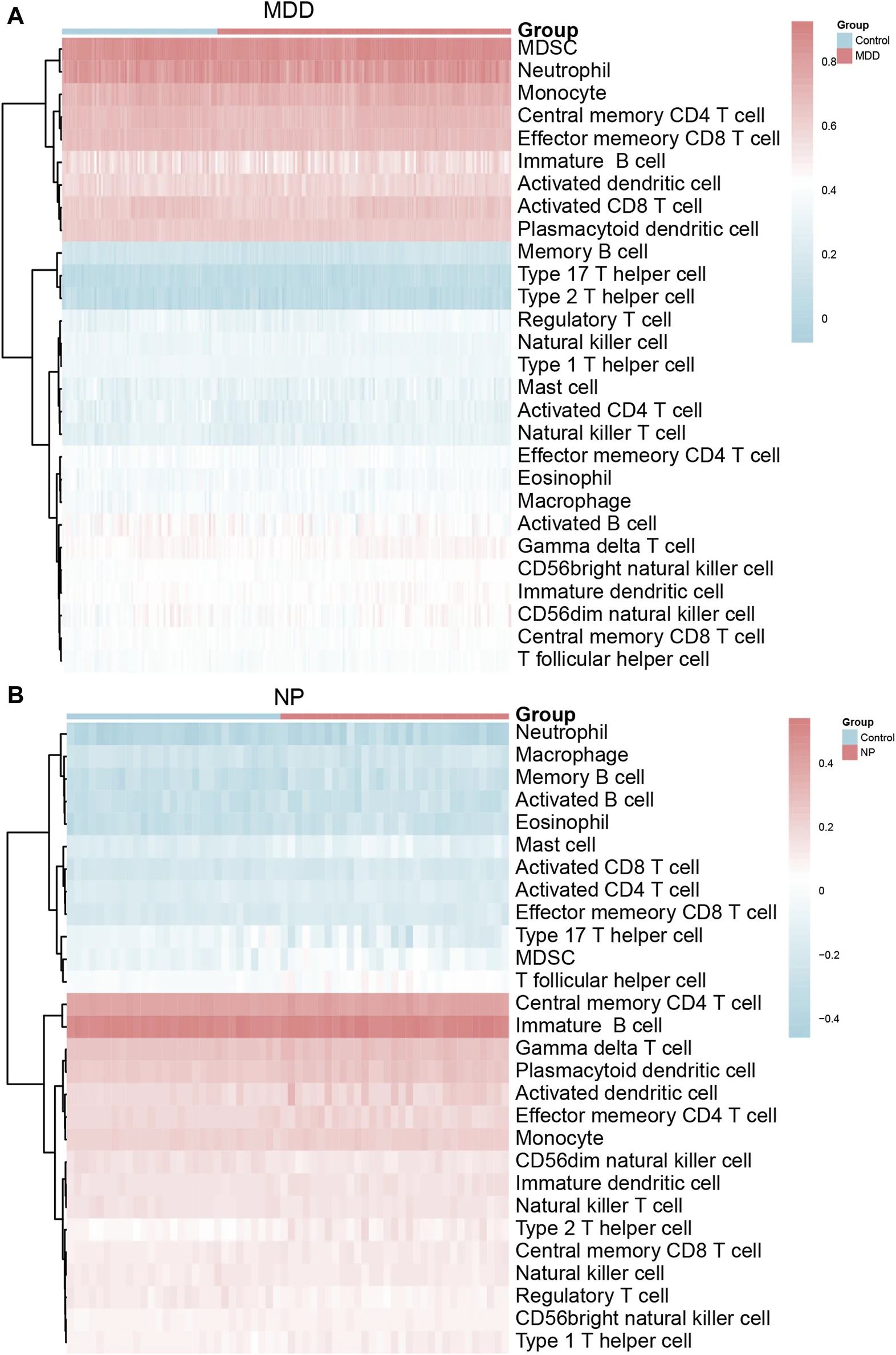
Immune characteristics difference analysis of MDD datasets samples and NP datasets samples. **(A,B)** Complex heatmap of ssGSEA immunoinfiltration analysis results for MDD datasets samples **(A)** and NP datasets samples **(B)** MDD, major depressive disorder; NP, Neuropathic Pain; ssGSEA: single-sample gene-set enrichment analysis.

**FIGURE 9:**
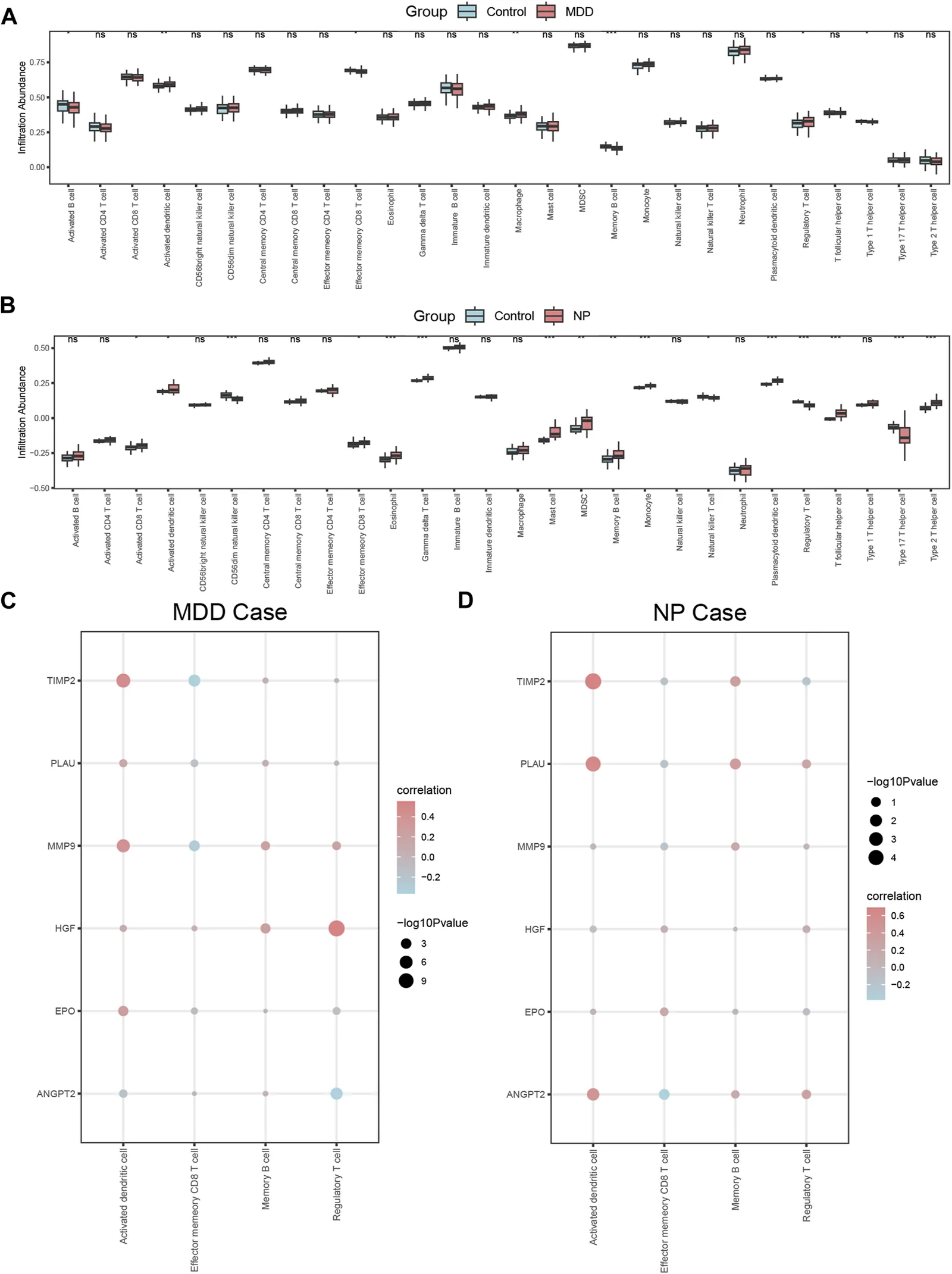
Correlation analysis of immune characteristics of MDD datasets disease samples and NP datasets disease samples. **(A,B)** Complex heatmap of ssGSEA immunoinfiltration analysis results for MDD datasets samples **(A)** and NP datasets samples **(B)** MDD, major depressive disorder; NP, Neuropathic Pain; ssGSEA: single-sample gene-set enrichment analysis. **(A,B)** Grouped comparison plot of ssGSEA immunoinfiltration analysis results for MDD datasets samples **(A)** and NP datasets samples **(B)**. **(C,D)** Correlation heat map of immune cell infiltration abundance and Co-hub genes expression in MDD datasets disease samples **(C)** and NP datasets disease samples **(D)**. The symbol ns is equivalent to *p* ≥ 0.05, which is not statistically significant; The symbol * is equivalent to *p* < 0.05; the symbol ** is equivalent to *p* < 0.01; and the symbol *** is equivalent to *p* < 0.001. MDD, major depressive disorder; NP, Neuropathic Pain; ssGSEA, single-sample gene-set enrichment analysis.

The relative infiltration of 28 immune cell types in the disease group (Case/MDD/NP) and control group samples in the MDD and NP dataset expression matrix were determined using the ssGSEA algorithm. We presented the findings for both datasets using a complex heat map (Figures 8A, B). The results indicated that among the disease and control group samples of MDD (Figure 8A) and NP (Figure 8B), the infiltration by the 28 cell types varied significantly. Next, we used the Mann-Whitney *U* test to analyze the differential degree of infiltration of these cells among the different groups (Case/Control) in the two datasets and the results are grouped in comparison plots (Figures 9A, B). The control group exhibited a higher abundance of activated B cells, effector memory CD8 T cells, memory B cells, and type 1 T helper cells compared with the disease group. In contrast, the disease group contained a more activated dendritic cells, macrophages, and regulatory T cells compared with the control group (Figure 9A).

CD56dim natural killer cells, natural killer T cells, regulatory T cells, and type 17 T helper cells had higher infiltration in the NP dataset control group compared with that in the disease group. In contrast, the disease group had higher infiltration of activated CD8 T cells, activated dendritic cells, effector memory CD8 T cells, eosinophils, gamma delta T cells, mast cells, MDSCs, memory B cells, monocyte neutrophils, plasmacytoid dendritic cells, T follicular helper cells, and type 2 T helper cells (Figure 9B).

Figures 9A, B shows that there were statistically significant differences (*p* < 0.05) between the relative immune infiltration of the MDD and NP dataset samples compared with control group (Case/Control) samples for activated dendritic cells, effector memory CD8 T cells, memory B cells, and regulatory T cells.

We also calculated the correlation for these 4 immune cell types with the expression of the 6 Co-hub genes (ANGPT2, EPO, HGF, MMP9, PLAU, TIMP2) in the MDD and NP dataset disease samples (Figures 9C, D). The results indicated that the expression of these 6 genes and the relative abundance of the four immune cells tended to be significantly positive and less negatively correlated (*p* < 0.05) in the MDD dataset samples (Figure 9C). The results of the correlation analysis between the infiltration of the 4 immune cells and the expression of 6 the Co-hub genes in the NP dataset disease samples revealed that there was a significant positive correlation (*p* < 0.05) between these infiltrating cells and the 6 genes. Activated dendritic cells showed the highest correlation with the expression of these genes (Figure 9D).

Finally, the expression of activated dendritic immune cells, memory B cells and the 6 Co-hub genes had a significant positive correlation in the MDD and NP dataset disease samples (Figures 9C, D). In contrast, the expression of the 6 Co-hub genes was significantly negatively correlated with the infiltration abundance of effector memory CD8 T immune cells in these datasets (Figures 9C, D).

### Correlation analysis of the Co-hub genes

**FIGURE 10:**
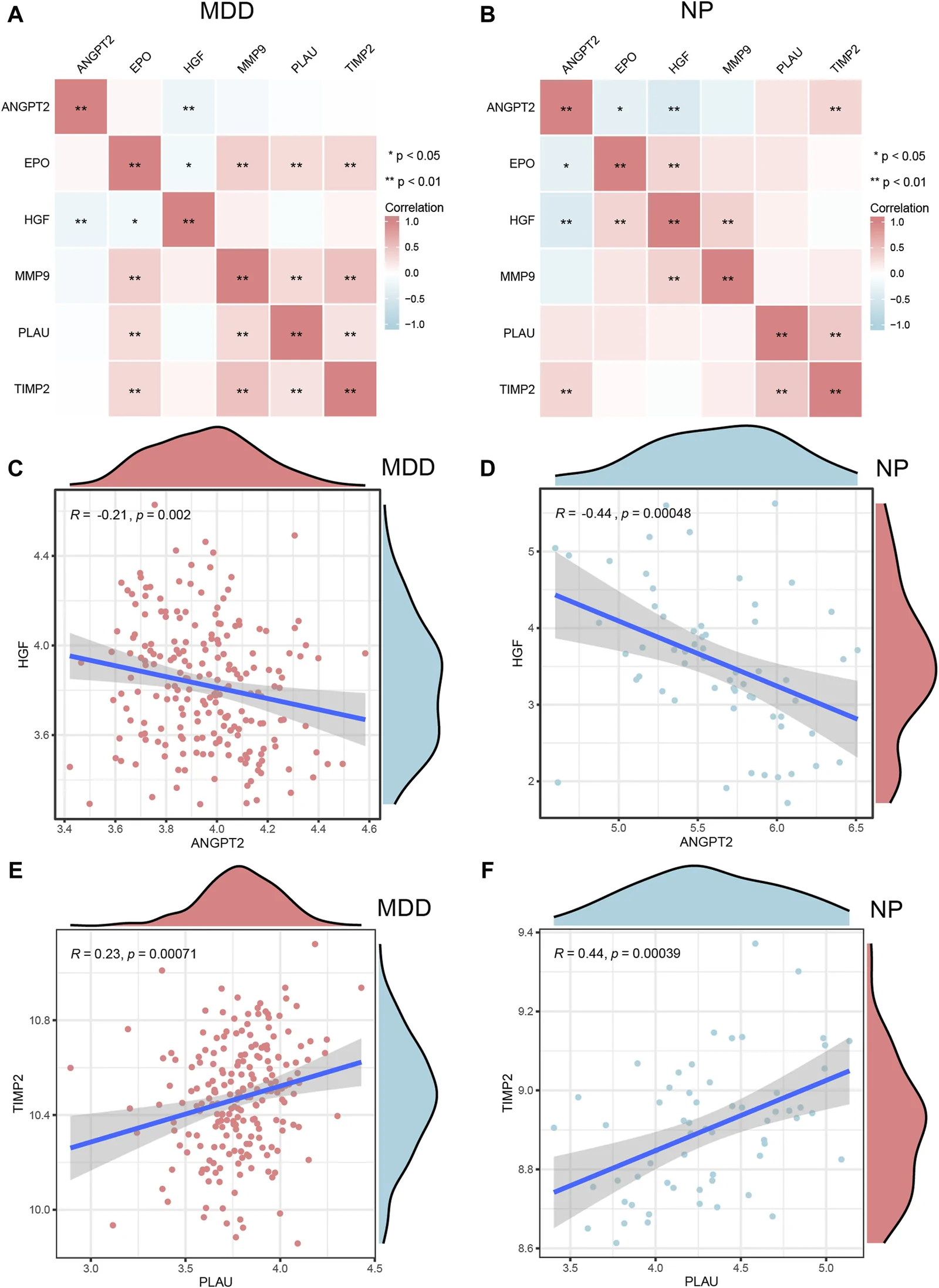
Correlation analysis of Co-hub genes. **A,B,** The correlation heat map results of Co-hub genes in MDD datasets **(A)**, NP datasets **(B)**. **(C,D)** The correlation scatterplot results of Co-hub genes ANGPT2 and HGF in MDD datasets **(C)** and NP datasets **(D)**. **(E,F)** The correlation scatterplot results of Co-hub genes PLAU and TIMP2 in the MDD datasets **(E)** and NP datasets **(F)**. *p* ≥ 0.05, not statistically significant; *p* < 0.05, statistically significant; *p* < 0.01, which was highly statistically significant; *p* < 0.001, which is exceedingly statistically significant. The correlation coefficient (r) in the correlation scatterplot is strongly correlated if the absolute value is above 0.8; the absolute value is 0.5–0.8 is moderately correlated; Absolute value of 0.3–0.5 is weakly correlated; absolute values below 0.3 are weak or uncorrelated. MDD, major depressive disorder; NP, Neuropathic Pain.

The correlation in expression between the 6 Co-hub genes (ANGPT2, EPO, HGF, MMP9, PLAU, TIMP2) was analyzed in the MDD and NP datasets by Spearman correlation analysis and presented as a correlation heat map (Figures 10A, B). There was a positive correlation among most Co-hub genes in both datasets; however, quite a few correlations of Co-hub genes in the NP datasets did not show a statistical difference (*p* > 0.05) (Figures 10A, B).

We selected interaction pairs that were statistically different (*p* < 0.05) with the same trend in the MDD and NP datasets for further analysis. Based on the Spearman algorithm, we generated scatter plots to show the correlation analysis results for the Co-hub genes, ANGPT2 and HGF, in the MDD and NP datasets as well as the correlation analysis result of PLAU and TIMP2 in the two datasets (Figures 10C–F). Among them, there were negative correlations between the expression of ANGPT2 and HGF (R = −0.21, Figure 10C), (R = 0.44, Figure 10D) in both datasets. Conversely, the expression of PLAU and TIMP2 (R = 0.23, Figure 10E), (R = 0.44, Figure 10F) had a positive correlation in both datasets.

### Differential expression analysis of the Co-hub genes

**FIGURE 11:**
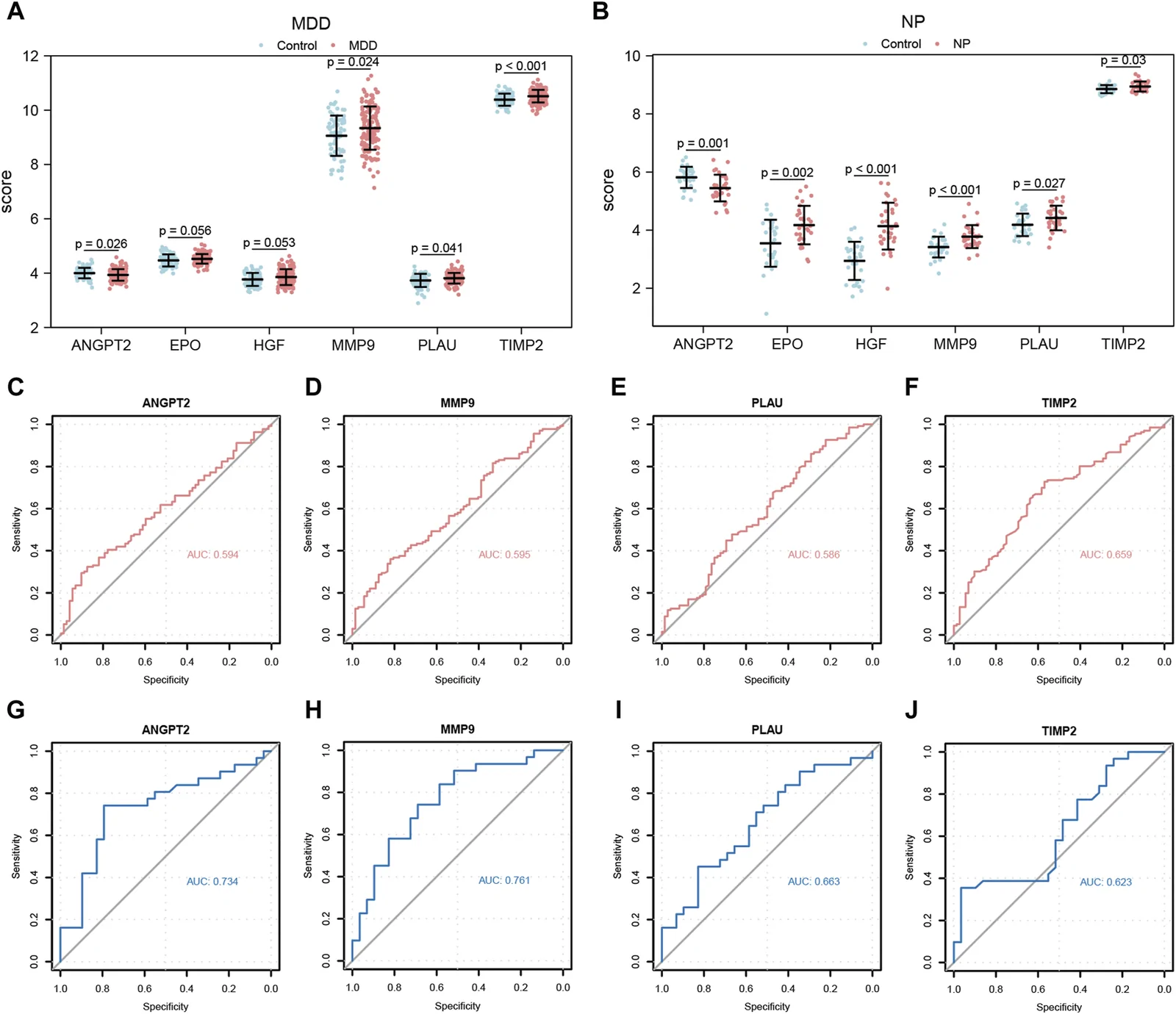
Expression differences analysis of Co-hub genes in MDD datasets and NP datasets. **(A,B)** The correlation heat map results of Co-hub genes in MDD datasets **(A)**, NP datasets **(B)**. **(C,D)** The correlation scatterplot results of Co-hub genes ANGPT2 and HGF in MDD datasets **(C)** and NP datasets **(D)**. **(E,F)** The correlation scatterplot results of Co-hub genes PLAU and TIMP2 in the MDD datasets **(E)** and NP datasets **(F)**. *p* ≥ 0.05, not statistically significant; *p* < 0.05, statistically significant; *p* < 0.01, which was highly statistically significant; *p* < 0.001, which is exceedingly statistically significant. The correlation coefficient (r) in the correlation scatterplot is strongly correlated if the absolute value is above 0.8; the absolute value is 0.5-0.8 is moderately correlated; Absolute value of 0.3-0.5 is weakly correlated; absolute values below 0.3 are weak or uncorrelated. MDD, major depressive disorder; NP, Neuropathic Pain. **(A,B)** Grouped comparison plot of Co-hub genes in MDD datasets **(A)**, NP datasets **(B)** in different groups (Case/Control). **(C–F)** ROC curves of Co-hub genes ANGPT2 **(C)**, MMP9 **(D)**, PLAU **(E)**, TIMP2 **(F)** in different groups of the MDD datasets. **(G–J)** ROC curves of Co-hub genes ANGPT2 **(G)**, MMP9 **(H)**, PLAU **(I)**, TIMP2 **(J)** in different groups of NP datasets. *p* ≥ 0.05, not statistically significant; *p* < 0.05, statistically significant; *p* < 0.01, which was highly statistically significant; *p* < 0.001, which is exceedingly statistically significant. The closer the AUC in the ROC curve is to 1, the better the diagnosis. AUC has low accuracy at 0.5~0.7; AUC has a certain accuracy at 0.7~0.9; AUC has high accuracy above 0.9. MDD, major depressive disorder; NP, Neuropathic Pain; ROC, receiver operating characteristic curve; AUC, Area Under the Curve.

For both datasets, we also examined the expression differences for the 6 Co-hub genes (ANGPT2, EPO, HGF, MMP9, PLAU, and TIMP2) between the disease group (group: Case/MDD/NP) and the control group (group: Control). The results are presented through grouped comparison plots (Figures 11A, B). Of the 6 common hub genes, only ANGPT2, MMP9, PLAU, and TIMP2 were statistically significantly different (*p* < 0.05) in the different groups of the MDD datasets. Although MMP9, PLAU, and TIMP2 expression were all significantly higher in the disease group of the MDD datasets compared with the control group, ANGPT2 expression was significantly lower in the disease group compared with the control group (Figure 11A). The 6 common hub genes in the NP datasets were all statistically significantly (*p* < 0.05) different in the different groups. Among them, ANGPT2 gene expression was upregulated in the normal group, whereas EPO, HGF, MMP9, PLAU, and TIMP2 were upregulated in the disease group (Figure 11B).

Next, we plotted the ROC curves for the four Co-hub genes (ANGPT2, MMP9, PLAU, TIMP2) in both datasets (Figures 11C–J). As shown in Figures 11C–F, the expression of ANGPT2 (AUC = 0.594, Figure 11C), MMP9 (AUC = 0.595, Figure 11D), PLAU (AUC = 0.586, Figure 11E), and TIMP2 (AUC = 0.659, Figure 11F) all had low accuracy for the diagnosis of MDD disease, whereas the expression of the Co-hub genes, ANGPT2 (AUC = 0.734, Figure 11G) and MMP9 (AUC = 0.761, Figure 11H), exhibited a certain accuracy for the diagnosis of NP disease. In contrast, the expression of PLAU (AUC = 0.663, Figure 11I) and TIMP2 (AUC = 0.623, Figure 11J) all had low accuracy for the diagnosis of NP disease.

### Differential expression analysis of the Co-hub genes in independent datasets

**FIGURE 12:**
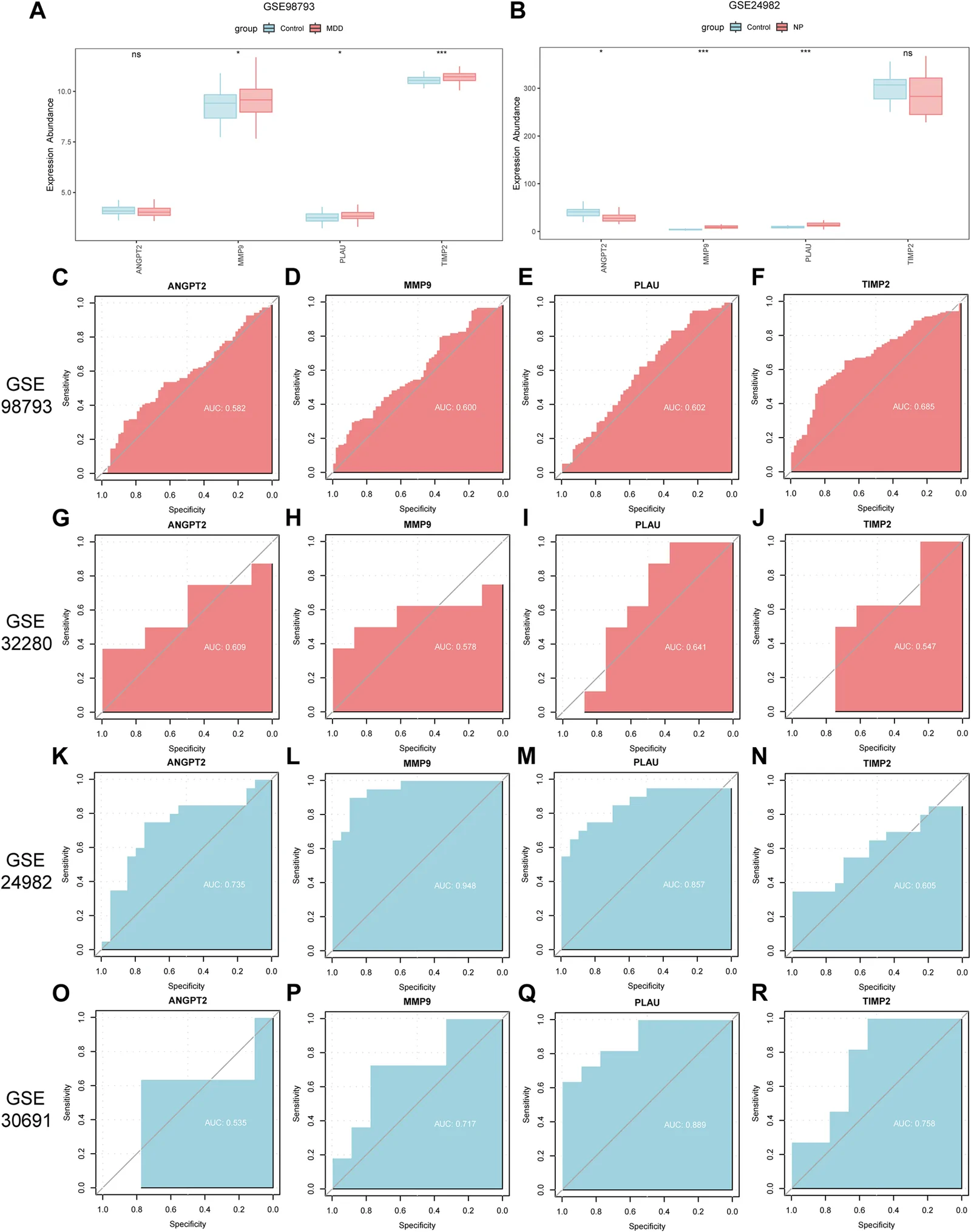
Differential expression analysis of co-hub genes in independent disease datasets. **(A,B)** Group comparison charts of co-hub genes in different groups (Case/Control) in the GSE98793 dataset **(A)** and the GSE24982 dataset **(B)**. **(C–F)** ROC curves of ANGPT2 **(C)**, MMP9 **(D)**, PLAU **(E)**, and TIMP2 **(F)** in different groups of the GSE98793 dataset. **(G–J)** ROC curves of ANGPT2 **(G)**, MMP9 **(H)**, PLAU **(I)**, and TIMP2 **(J)** in different groups of the GSE32280 dataset. **(K–N)** ROC curves of ANGPT2 **(K)**, MMP9 **(L)**, PLAU **(M)**, and TIMP2 **(N)** in different groups of the GSE24982 dataset. **(O–R)** ROC curves of ANGPT2 **(O)**, MMP9 **(P)**, PLAU **(Q)**, and TIMP2 **(R)** in different groups of the GSE30691 dataset. The symbol ns is equivalent to *p* ≥ 0.05, without statistical significance; the symbol * is equivalent to *p* < 0.05, with statistically significant meaning; the symbol ** is equivalent to *p* < 0.01, with highly statistically significant meaning; the symbol *** is equivalent to *p* < 0.001, with extremely statistically significant meaning. In the ROC curve, the closer the AUC is to 1, the better the diagnostic effect. When the AUC is between 0.5 and 0.7, there is low accuracy; when the AUC is between 0.7 and 0.9, there is certain accuracy; when the AUC is above 0.9, there is high accuracy. MDD, major depressive disorder; NP, Neuropathic Pain; ROC, receiver operating characteristic curve; AUC, Area Under the Curve.

To verify the expression differences of 4 co-hub genes (ANGPT2, MMP9, PLAU, TIMP2) in MDD and NP datasets, we also used the Wilcoxon rank sum test to analyze the expression levels of these 4 co-hub genes in GSE98793 and GSE24982 datasets. The differences between the disease group (group: Case/MDD/NP) and the control group (group: Control) were shown through comparison charts (Figures 12A, B). As shown in Figures 12A, 11B, MMP9, PLAU, and TIMP2 all had statistically significant differences (*p* < 0.05) in different groups of the MDD datasets. The expression levels of MMP9, PLAU, and TIMP2 in the disease group of GSE98793 dataset were significantly higher than those in the control group (Figure 12A). In the NP dataset, ANGPT2, MMP9, and PLAU all had statistically significant differences (*p* < 0.05) between different groups. The expression level of ANGPT2 was upregulated in the control group while the expression levels of MMP9 and PLAU were upregulated in the disease group (Figure 12B).

We then plotted the ROC curves of the 4 co-hub genes (ANGPT2, MMP9, PLAU, TIMP2) in GSE98793, GSE32280, GSE24982 and GSE30691 datasets and presented the results (Figures 12C–R). As shown in Figures 12C–F, the expression of co-hub genes ANGPT2 (AUC = 0.582, Figure 12C), MMP9 (AUC = 0.600, Figure 12D), PLAU (AUC = 0.602, Figure 12E), and TIMP2 (AUC = 0.685, Figure 12F) all had low accuracy in diagnosing MDD in the GSE98793 dataset. Similarly, as shown in Figures 12G–J, the expression of co-hub genes ANGPT2 (AUC = 0.609, Figure 12G), MMP9 (AUC = 0.578, Figure 12H), PLAU (AUC = 0.641, Figure 12I), and TIMP2 (AUC = 0.547, Figure 12J) all had low accuracy in diagnosing MDD in the GSE32280 dataset.

As shown in Figures 12K–N, the expression of co-hub gene MMP9 (AUC = 0.948, Figure 12L) had high accuracy in diagnosing NP in the GSE24982 dataset. The expression of ANGPT2 (AUC = 0.735, Figure 12K) and PLAU (AUC = 0.857, Figure 12M) had some accuracy in diagnosing NP in the GSE24982 dataset while the expression of TIMP2 (AUC = 0.605, Figure 12N) had low accuracy in diagnosing NP in the GSE24982 dataset.

As shown in Figures 12O–R, the expression of common hub genes MMP9 (AUC = 0.717, Figure 12P), PLAU (AUC = 0.889, Figure 12Q), and TIMP2 (AUC = 0.758, Figure 12R) had some accuracy in diagnosing NP in the GSE30691 dataset while the expression of ANGPT2 (AUC = 0.535, Figure 12O) had low accuracy in diagnosing NP in the GSE30691 dataset. Finally, we summarized the clinical information of different groups in the GSE98793 dataset and presented the results in a clinical data table (Supplementary Table S3).

## Discussion

NP is a common chronic pain with a prevalence ranging from 6.9% to 10.0% [3], which significantly reduces the quality of life for individuals [40]. Approximately 50% of NP patients report having depression [41] and when NP patients are co-depressed, they have poor physical, and psychological functioning, which results in persistent physical and mental distress [42]. Depression and NP are often accompanied by many overlapping symptoms, and antidepressants are used to treat NP, suggesting that they share many common neural circuits and underlying mechanisms, such as neuroinflammation [43]. Studies have found a correlation between the incidence of NP and depression [44] determining whether NP, and depression share a common pathological and molecular mechanism is important for clinical diagnosis and treatment. An understanding of the molecular pathways of disease initiation and development using microarray and bioinformatics analysis will enable us to examine genetic variations and discover novel diagnostic biomarkers and therapeutic targets.

However, when a single dataset is analyzed, one-sided results may be obtained, resulting in a higher false-positive rate. Therefore, in the present study, we combined two datasets for MDD (GSE98793, GSE32280) and two datasets for NP (GSE24982, GSE30691). The combined MDD datasets contained 136 cases and 72 control samples, whereas the NP datasets contained 31 cases and 29 control samples. In total, after analyzing both datasets, we identified 6 Co-hub genes, which included ANGPT2, EPO, HGF, MMP9, PLAU, and TIMP2. The results indicated that ANGPT2, MMP9, PLAU, and TIMP2 expression variations in both datasets were statistically significant. The ROC curves revealed that ANGPT2 and MMP9 can diagnose NP with some accuracy. In addition, we found that the abundance of infiltrating activated dendritic cells, effector memory CD8^+^ T cells, memory B cells, and regulatory T cells changed significantly (*p* < 0.05).

The functional similarity analysis results between the 6 Co-hub genes indicated that HGF had the highest value compared with the other Co-hub genes (Figure 7C). HGF is a protein-coding gene that acts as a growth factor by promoting hepatocyte regeneration in stem and progenitor cells, which is activated by binding to the c-MET receptor [45]. As a neurotrophic factor, HGF/c-MET is essential for the growth of axons, the development of the central nervous system, and the defense of neurons [46]. In previous mice studies, HGF was demonstrated to improve the symptoms of NP and induce functional recovery and regeneration of neurons [47–50]. By regulating crucial elements linked to DRG neuropathic pain and lowering the spinal microglia activity, HGF produces analgesic effects. Additionally, in a cross-sectional, multicenter investigation of diabetic individuals with neuropathic pain, HGF was linked to increased pain levels [51]. In MDD, psychiatric symptoms, such as anxiety and depression, are also significantly associated with cerebrospinal fluid HGF levels [52]. Downregulation of HGF/c-MET signaling in the hippocampus may be associated with methylation alterations in MET during MDD pathophysiology [53]. Several studies on depression across various groups have also demonstrated a significant relationship between changes in HGF levels and depression [54, 55], and HGF may be useful in assessing the severity of depression-related symptoms [56]. Combined with our results, neuropathic pain, and melancholy conditions may be significantly influenced by HGF. In both the MDD and NP datasets, we found a negative association between the expression of ANGPT2 and HGF (Figures 10C,D). ANGPT2 belongs to the angiopoietin family of growth factors that are upregulated in a variety of inflammatory diseases and are associated with direct control of inflammation-related signaling pathways. Based on our results, the expression of ANGPT2 has a low accuracy for the diagnosis of MDD (Figure 11C) and a certain degree of accuracy for the diagnosis of NP (Figure 11G); however, there are currently no studies related to ANGPT2 in NP or MDD, thus we are the first to discover that ANGPT2 may also play a key role in these diseases.

In numerous models of central and peripheral nerve damage, EPO exhibits a variety of neuroprotective benefits [57, 58]. EPO can alleviate neuropathic pain brought on by peripheral nerve damage by regulating the production of AQP-2 through the AMPK/mTOR/p70S6K pathway [59]. EPO and non-erythropoietic derivatives have also shown potential pro-cognitive effects in psychiatric disorders [60]. The non-erythropoietic derivative ARA290 can reduce inflammation and depression, which prolongs stress in rodents [61]. Studies have found that MDD may be related to neuronal plasticity damage8. Additionally, the neurotrophic and neuroprotective benefits of EPO and brain-derived neurotrophic factor (BDNF) can restore neural plasticity [62]. EPO and non-erythropoietic compounds, such as carbamoylated EPO, increase the production of BDNF in the hippocampus of rats [63]. Additional evidence indicating that EPO acts on the brain through neurotrophic and synaptic plasticity mechanisms has been derived from a bioinformatics study [64]. These studies are consistent with the results of our data mining in which EPO is a Co-hub gene for NP and MDD.

PLAU is a serine protease that converts plasminogen into plasmin, which is crucial for breaking down the extracellular matrix and encouraging fibrinolysis [65]. UPA, a PLAU expression product, can activate or suppress the inflammatory reaction through the AMPK and PI3K/Akt pathways [66]. A previous study identified PLAU as a hub gene of NP [67], which is consistent with our results; however, no studies have associated PLAU with MDD. Our study is the first to show that PLAU expression has low accuracy for the diagnosis of both NP and MDD (Figures 11I,E).

We found that MMP9 expression had a certain accuracy for the diagnosis of NP disease and low accuracy for the diagnosis of MDD (Figures 11D,H). In addition, TIMP2 expression has low accuracy for the diagnosis of NP and MDD occurrence (Figures 11J, 12F). It also plays a role in angiogenesis, axon growth, and neuroplasticity [68, 69]. MMP9, as a mediator of neuroinflammation, influences the onset and progression of NP by stimulating DRG and microglia in the spinal cord, participates in the regulation of oxidative stress and the inflammatory response, affects the maturation of inflammatory cytokines and may be directly involved in the development and maintenance of NP [70]. MMP9 may also cause a proBDNF/mBDNF (brain-derived neurotrophic factor) imbalance by influencing the process by which proBDNF is converted into mBDNF, leading to depression [71]. Decreased MMP9 levels result in decreased neuronal differentiation in the hippocampus and may cause increased anxiety in mice [72]. Four TIMPs(TIMP1, TIMP2, TIMP3, TIMP4) are physiological tissue inhibitors of MMPs, of which TIMP2 can indirectly affect NP and MDD processes by inhibiting MMP9 activity [73, 74]. These studies confirm the results of our database analysis that MMP9 and TIMP2 have some diagnostic value for NP and MDD.

Our finding that immune cells are associated with the emergence, maintenance, and cure of NP and MDD is consistent with previous studies [75–77]. As shown in Figures 9A,B, the difference in abundance of infiltrating immune cells, including activated dendritic cells, effector memory CD8^+^ T cells, memory B cells, and regulatory T cells, in the disease, and control group of the MDD datasets and NP datasets was statistically significant. It is worth noting that, the infiltration abundance of Activated dendritic cells is significantly increased in both NP and MDD disease groups. However, the infiltration abundance of Effector memory CD8^+^ T cells and Memory B cells is significantly increased in the NP disease group, but significantly decreased in the MDD disease group. Conversely, the infiltration abundance of Regulatory T cells is significantly decreased in the NP disease group, but significantly increased in the MDD disease group. Effector memory CD8^+^ T cells are a type of memory T cell that can rapidly respond to re-infection and are capable of secreting cytokines and killing target cells [78]. Activated dendritic cells are dendritic cells that have captured and processed antigens and have undergone phenotypic and functional changes [79], they are able to more effectively activate T cells and secrete a variety of cytokines to regulate immune responses. Activated dendritic cells and effector memory CD8^+^ T cells were found to be significantly upregulated in NP [80], which is consistent with our findings. Sun et al. found that the proportion of CD8^+^ T cells in MDD patients is low, and they are divided into 2 subtypes: a subtype with a higher proportion of CD8^+^ T cells and a subtype with a lower proportion of CD8^+^ T cells. In the subtype with a higher proportion of CD8^+^ T cells, the expression levels of genes related to autophagy, immune response, and apoptosis are higher. Reducing the apoptosis of CD8^+^ T lymphocytes can reduce the level of inflammatory factors and improve the immune microenvironment of depressed mice [81]. These results suggest that effector memory CD8^+^ T cells may play an important role in the pathogenesis of NP and MDD. In this study, activated dendritic cells had the highest infiltration associated with the expression of key genes (Figure 9D). Maganin et al. found that dendritic cells cause NP by promoting the kynurenine metabolic pathway [82]. Wang et al. concluded that dendritic cells cause NP by sensitizing nociceptor sensory neurons through paracrine factors [83]. Stiglbauer et al. found that obesity and MDD patients have fewer dendritic cells and effector memory CD8^+^ T cells compared with normal-weight patients who were not depressed [84]. Ciaramella et al. found that the decrease in dendritic cells is associated with the severity of depressive symptoms in Alzheimer’s disease patients [85]. These studies and our results consistently suggest that changes in the number and function of dendritic cells may be involved in the common pathophysiology of comorbid NP and MDD. Memory B cells are formed within the germinal center after the primary infection and play an important role in the secondary immune response [86]. Combining Figures 9C, D, it is evident that the expression of activated dendritic cells, memory B cells, and the 6 Co-hub genes are positively correlated in the MDD and NP datasets disease samples. A previous bioinformatics analysis also found that memory B cells correlated with MDD diagnostic marker genes [87]. The role of memory B cells in NP is currently unclear, and there is no rigorous evidence to show the relationship between memory B cells and NP. The discovery of increased infiltration abundance of memory B cells in NP is a new finding in this study, and further research is needed to explore the role of memory B cells in the comorbidity of NP and MDD. Regulatory T cells control the body’s immune response to harmful invaders and prevent overreaction [88]. A study indicated that regulatory T -cells can prevent pain-induced hypersensitivity reactions caused by microglia [89]. In depressed patients, there is a decrease in regulatory T cells [76]. Taken together, there is an immune-activated microenvironment in NP and MDD comorbidities, and immunity, and inflammation may play an important role in NP and MDD comorbidities. Activated dendritic cells, effector memory CD8^+^ T cells, memory B cells, and regulatory T cells have the potential to be therapeutic targets for NP and MDD. However, articles researching the relationship between these immune cells and NP or MDD are very limited. Their specific roles in the comorbidity of NP and MDD are not clear, and more research is needed to explore the biological significance behind these immune changes.

There are some limitations to this study. First, the study integrates human whole blood samples and peripheral blood lymphocyte chip data on the same sequencing platform for analysis. While it can provide valuable information regarding cell type comparison, immune response analysis, cellular interactions, disease-related analysis, and the identification of potential biomarkers, contributing to a deeper understanding of immune system functionality and the mechanisms underlying related diseases, this approach also has limitations and challenges such as sample heterogeneity, differential gene expression, signal dilution, and technical variations. To overcome these limitations, future research can consider strategies such as cell sorting, single-cell sequencing, and experimental validation to help overcome these challenges and provide a more focused and accurate analysis of lymphocyte-specific gene expression changes. Secondly, compared to MDD, the sample size of NP is relatively low. In the future, the reliability of the results should be evaluated through hypothesis testing based on sample size, cross-validation, and other methods. Thirdly, the MDD sample is human-sourced, while the NP sample is animal-sourced, and this species difference may have an impact on the results’ generalizability. In addition, although batch-effect correction was performed, it is important to note that residual batch effects may still persist in the analysis due to variations in sample processing, experimental conditions, or other technical factors that could not be entirely eliminated. In the future, Emerging machine learning algorithms, such as deep neural networks, can also be used for data mining and analysis to better understand the relationships and trends between samples. Future studies should also explore the role of various immune cells in the co-morbid mechanisms of NP and MDD, and search for therapeutic targets of NP and MDD through anti-inflammatory pathways.

In conclusion, after screening 93 Co-DEGs, and performing GO and KEGG enrichment analyses, we identified 6 Co-hub genes, which included ANGPT2, EPO, HGF, MMP9, PLAU, and TIMP2. We also found that between the disease group and the control group for NP and MDD, there were significant differences in the abundance of activated dendritic cells, effector memory CD8^+^ T cells, memory B cells, and regulatory T cells. The possible diagnostic or therapeutic value of these immune cells and genes in NP and MDD warrant further study.

## Data Availability

The original contributions presented in the study are included in the article/Supplementary Material, further inquiries can be directed to the corresponding authors.
