# Correction: Bioinformatics and systems biology approach to identify the pathogenetic link of neurological pain and major depressive disorder

**DOI:** 10.3389/ebm.2026.11307

**Published:** 2026-10-26

**Authors:** Jinjing Hu, Jia Fu, Yuxin Cai, Shuping Chen, Mengjian Qu, Lisha Zhang, Weichao Fan, Ziyi Wang, Qing Zeng, Jihua Zou

**Affiliations:** 1 Department of Rehabilitation Medicine, Zhujiang Hospital, Southern Medical University, Guangzhou, China; 2 School of Rehabilitation Medicine, Southern Medical University, Guangzhou, China; 3 Department of Rehabilitation Sciences, Hong Kong Polytechnic University, Kowloon, Hong Kong SAR, China; 4 Department of Rehabilitation, The First Affiliated Hospital, Hengyang Medical School, University of South China, Hengyang, China; 5 Rehabilitation Laboratory, The First Affiliated Hospital, Hengyang Medical School, University of South China, Hengyang, China; 6 Faculty of Health and Social Sciences, Hong Kong Polytechnic University, Kowloon, Hong Kong SAR, China; 7 Department of Clinical Medicine, Suzhou Vocational Health College, Suzhou, China; 8 The First School of Clinical Medicine, Southern Medical University, Guangzhou, China

**Keywords:** neurological pain, gene expression omnibus (GEO), major depressive disorder (MDD), bioinformatics, receiver operating characteristic (ROC)

The abbreviation “MCC” was incorrectly expanded as “Matthews Correlation Coefficient metric” in the published article. The correct expansion is “Maximal Clique Centrality.”

A correction has been made to the section Materials and Methods, Protein-protein interaction network analysis, Paragraph 2:

“We also used four algorithms to mine the scores of Co-DEGs in PPI networks that are connected to other PPI network nodes, which included MCC (Maximal Clique Centrality) [33], MNC (the maximal neighborhood coefficient), EPC (edge percolated component), and Closeness. According to our rankings of the Co-DEGs, the top 20 Co-DEGs across the four algorithms were considered as hub genes (hub genes, mRNA).”

A correction has been made to the section Results, CytoHubba plug-in identifies the hub genes, Paragraph 1:

“We calculated the PPI network using the cytoHubba plugin for Cytoscape using four algorithms: MCC (Maximal Clique Centrality), MNC, EPC (edge percolated component), and Closeness. The top 20 Co-DEGs with the best scores were selected to further identify the hub genes in the Co-DEGs PPI network (**Figures 6A–D**). ”

A correction has been made to the **Figure 6** legend:

“FIGURE 6. CytoHubba plug-in node network for Co-DEGs PPI network and GO and KEGG enrichment analysis. The top 20 node network of the MCC **(A)**, MNC **(B)**, EPC **(C)**, Closeness **(D)** algorithm of the PPI network of Co-DEGs. **(E)** The top 20 nodes of the Venn diagram result from the four algorithms of MCC, MNC, EPC, and Closeness in the Co-DEGs PPI network. **(F)** The bubble plot of GO/KEGG enrichment analysis results of 8 hub genes. **(G)** The network diagram of GO enrichment analysis results of Co-DEGs. **(H)** The ring network diagram of KEGG pathway enrichment analysis results of Co-DEGs. The abscissa in the bubble chart **(F)** is GO/KEGG terms, and the length of the bubble distance from the X-axis represents the GeneRatio value of GO terms. Co-DEGs, Common differentially expressed genes; PPI network, Protein-protein interaction network; MCC, Maximal Clique Centrality; MNC, the maximal neighborhood coefficient; EPC, edge percolated component; GO, Gene Ontology; BP, biological process; CC, cellular component; MF, molecular function; KEGG, Kyoto Encyclopedia of Genes and Genomes. The screening criteria for GO/KEGG-enriched entries were p value <0.05 and FDR value (q.value) < 0.05.”

Reference 33 was incorrectly cited in the text. The correct reference is “Chin CH, Chen SH, Wu HH, Ho CW, Ko MT, Lin CY. cytoHubba: identifying hub objects and sub-networks from complex interactome. *BMC Syst Biol*. 2014; 8 Suppl 4(Suppl 4):S11. doi:10.1186/1752-0509-8-S4-S11”.

A correction has been made to the section Reference, 33: “Chin CH, Chen SH, Wu HH, Ho CW, Ko MT, Lin CY. cytoHubba: identifying hub objects and sub-networks from complex interactome. *BMC Syst Biol*. 2014; 8 Suppl 4(Suppl 4):S11. doi:10.1186/1752-0509-8-S4-S11”

The gene symbol “ANGPT” was used in three places where it was inconsistent with the surrounding context and with the gene symbol “ANGPT2” used elsewhere in the manuscript, which may cause misunderstanding.

A correction has been made to the section Results, MCODE plug-in hub-node analysis;

The PPI network of Co-hub genes; including the following four instances: “PLAU, TIMP2, HGF, ANGPT, MMP9, EPO” has been corrected to “PLAU, TIMP2, HGF, ANGPT2, MMP9, EPO.”

“The 9 (hub node) genes included PLAU, TIMP2, HGF, ANGPT, MMP9, EPO, RPL30, TTC37, and WDR61.” has been corrected to “The 9 (hub node) genes included PLAU, TIMP2, HGF, ANGPT2, MMP9, EPO, RPL30,TTC37, and WDR61.”

In paragraph 2 “On the 9 hub nodes (PLAU, TIMP2, HGF, ANGPT,MMP9, EPO, RPL30, TTC37, WDR61),”has been corrected to“On the 9 hub nodes (PLAU, TIMP2, HGF, ANGPT2,MMP9, EPO, RPL30, TTC37, WDR61),”.

The PPI network of Co-hub genes section, paragraph 1 “We first obtained Co-hub genes by taking the intersection of the 9 hub nodes (PLAU, TIMP2, HGF, ANGPT, MMP9, EPO, RPL30, TTC37, WDR61)” has been corrected to “We first obtained Co-hub genes by taking the intersection of the 9 hub nodes (PLAU, TIMP2, HGF, ANGPT2, MMP9, EPO, RPL30, TTC37, WDR61)”.

The abbreviation “MMD” was a typographical error in the Discussion section when referring to major depressive disorder. The correct abbreviation is “MDD” (major depressive disorder).

A correction has been made to the section Discussion, Paragraph 2:

“Therefore, in the present study, we combined two datasets for MMD (GSE98793, GSE32280) and two datasets for NP (GSE24982, GSE30691). The combined MMD datasets contained 136 cases and 72 control samples, whereas the NP datasets contained 31 cases and 29 control samples.” has been corrected to “Therefore, in the present study, we combined two datasets for MDD (GSE98793, GSE32280) and two datasets for NP (GSE24982, GSE30691). The combined MDD datasets contained 136 cases and 72 control samples, whereas the NP datasets contained 31 cases and 29 control samples.”

The name “ControlizeBetweenArrays” was incorrectly reported in the Methods section under “Editing of raw data.” The correct function for between-array normalization is “normalizeBetweenArrays” with the quantile normalization method.

A correction has been made to the section Materials and Methods, Editing of raw data:

“First standardized internally using the ControlizeBetweenArrays function of the R limma package” has been corrected to “first standardized internally using the normalizeBetweenArrays function of the R limma package with the quantile normalization method.”

The original version of this article has been updated.

