# Supplementary material for: Bioinformatics and systems biology approach to identify the pathogenetic link of neurological pain and major depressive disorder
Source: Exp Biol Med (Maywood). 2024 Jun 27;249:10129. doi: 10.3389/ebm.2024.10129 (PMC11236560; doi:10.3389/ebm.2024.10129)
Supplement: Supplementary file 1 [file DataSheet1.pdf]

Supplementary Tables

Table S1. GO and KEGG enrichment analysis results of 9 hub node genes.

Ontology	ID	Description	GeneRatio	BgRatio	pvalue	p.adjust	qvalue
BP	GO:2001234	negative regulation of apoptotic signaling pathway	3/9	230/18670	1.47e-04	0.012	0.006
BP	GO:0001666	response to hypoxia	3/9	359/18670	5.43e-04	0.013	0.006
BP	GO:2001233	regulation of apoptotic signaling pathway	3/9	406/18670	7.78e-04	0.013	0.006
BP	GO:0002283	neutrophil activation involved in immune response	3/9	488/18670	0.001	0.017	0.008
BP	GO:0007568	aging	2/9	321/18670	0.010	0.047	0.022
CC	GO:0070820	tertiary granule	3/9	164/19717	4.57e-05	0.001	4.36e-04
CC	GO:0035327	transcriptionally active chromatin	2/9	25/19717	5.53e-05	0.001	4.36e-04
CC	GO:1904813	ficolin-1-rich granule lumen	2/9	124/19717	0.001	0.014	0.005
CC	GO:0034774	secretory granule lumen	2/9	321/19717	0.009	0.044	0.016
CC	GO:0060205	cytoplasmic vesicle lumen	2/9	338/19717	0.010	0.044	0.016
MF	GO:0004252	serine-type endopeptidase activity	3/9	160/17697	5.85e-05	8.85e-04	5.46e-04
MF	GO:0008236	serine-type peptidase activity	3/9	182/17697	8.59e-05	8.85e-04	5.46e-04
MF	GO:0017171	serine hydrolase activity	3/9	186/17697	9.16e-05	8.85e-04	5.46e-04
MF	GO:0004175	endopeptidase activity	3/9	427/17697	0.001	0.008	0.005
MF	GO:0008191	metalloendopeptidase inhibitor activity	1/9	16/17697	0.008	0.047	0.029
KEGG	hsa05205	Proteoglycans in cancer	3/8	205/8076	8.21e-04	0.028	0.019

Ontology	ID	Description	GeneRatio	BgRatio	pvalue	p.adjust	qvalue
KEGG	hsa03018	RNA degradation	2/8	79/8076	0.003	0.033	0.022
KEGG	hsa05215	Prostate cancer	2/8	97/8076	0.004	0.033	0.022
KEGG	hsa04151	PI3K-Akt signaling pathway	3/8	354/8076	0.004	0.033	0.022
KEGG	hsa04066	HIF-1 signaling pathway	2/8	109/8076	0.005	0.033	0.022

GO, Gene Ontology; BP, biological process; CC, cellular component; MF, molecular function; KEGG, Kyoto Encyclopedia of Genes and Genomes.

Table S2. GO and KEGG enrichment analysis results of 8 hub genes.

Ontology	ID	Description	GeneRatio	BgRatio	pvalue	p.adjust	qvalue
BP	GO:0001666	response to hypoxia	4/8	359/18670	8.85e-06	0.001	5.05e-04
BP	GO:0030225	macrophage differentiation	2/8	44/18670	1.51e-04	0.006	0.002
BP	GO:2001233	regulation of apoptotic signaling pathway	3/8	406/18670	5.27e-04	0.010	0.004
BP	GO:0050727	regulation of inflammatory response	3/8	485/18670	8.85e-04	0.012	0.004
BP	GO:0007568	aging	2/8	321/18670	0.008	0.033	0.012
CC	GO:0070820	tertiary granule	3/8	164/19717	3.07e-05	8.29e-04	4.20e-04
CC	GO:1904813	ficolin-1-rich granule lumen	2/8	124/19717	0.001	0.010	0.005
CC	GO:0034774	secretory granule lumen	2/8	321/19717	0.007	0.026	0.013
CC	GO:0060205	cytoplasmic vesicle lumen	2/8	338/19717	0.008	0.026	0.013
CC	GO:0062023	collagen-containi ng extracellular matrix	2/8	406/19717	0.011	0.033	0.017
MF	GO:0004252	serine-type endopeptidase activity	3/8	160/17697	3.93e-05	8.41e-04	3.45e-04
MF	GO:0005125	cytokine activity	2/8	220/17697	0.004	0.024	0.010
MF	GO:0070700	BMP receptor binding	1/8	11/17697	0.005	0.025	0.010
MF	GO:0039706	co-receptor binding	1/8	13/17697	0.006	0.025	0.010
MF	GO:0005126	cytokine receptor binding	2/8	286/17697	0.007	0.025	0.010
KEGG	hsa05202	Transcriptional misregulation in cancer	3/7	192/8076	4.32e-04	0.010	0.007
KEGG	hsa05205	Proteoglycans in cancer	3/7	205/8076	5.23e-04	0.010	0.007
KEGG	hsa04151	PI3K-Akt signaling pathway	3/7	354/8076	0.003	0.027	0.019

Ontology	ID	Description	GeneRatio	BgRatio	pvalue	p.adjust	qvalue
KEGG	hsa05215	Prostate cancer	2/7	97/8076	0.003	0.027	0.019
KEGG	hsa04066	HIF-1 signaling pathway	2/7	109/8076	0.004	0.027	0.019

GO, Gene Ontology; BP, biological process; CC, cellular component; MF, molecular function; KEGG, Kyoto Encyclopedia of Genes and Genomes.

Table S3. Patient Characteristics of GSE98793 datasets.

Characteristics	Control	MDD
n	64	128
Gender, n (%)		
Male	16 (8.3%)	32 (16.7%)
Female	48 (25%)	96 (50%)
Age, median (IQR)	52.55 (44.05, 62.05)	52.65 (43.775, 62.05)
Anxiety, n (%)		
no	64 (33.3%)	64 (33.3%)
yes	0 (0%)	64 (33.3%)
